# A novel approach employing hesitant intuitionistic fuzzy linguistic Einstein aggregation operators within the EDAS approach for multicriteria group decision making

**DOI:** 10.1016/j.heliyon.2024.e31407

**Published:** 2024-05-22

**Authors:** Shahzad Faizi, Wojciech Sałabun, Mubashar Shah, Tabasam Rashid

**Affiliations:** a Department of Mathematics, Virtual University of Pakistan, Pakistan; b West Pomeranian University of Technology in Szczecin, Poland; c Department of Mathematics, University of Management and Technology, Lahore 54770, Pakistan

**Keywords:** Hesitant fuzzy set (HFS), Hesitant fuzzy linguistic term set (HFLTS), Hesitant intuitionistic fuzzy linguistic term set (HIFLTS), Multicriteria group decision making (MCGDM), The evaluation based on distance from average solution (EDAS)

## Abstract

The Evaluation based on Distance from Average Solution (EDAS) is a multi-criteria decision analysis (MCDA) technique that uses various distances from average values to make decisions. It bears resemblance to other distance-based approaches like SPOTIS, VIKOR or TOPSIS, except that instead of positive and negative ideal solutions, it uses an average solution. For hesitant intuitionistic fuzzy linguistic term sets (HIFLTSs), we first define several operational laws and aggregation operators. As a follow-up, the hesitating intuitionistic fuzzy linguistic Einstein weighted averaging (HIFLEWA) operator is introduced. In this study, the EDAS technique is used to address a multi-criteria group decision making (MCGDM) issue by utilizing the suggested operational laws and aggregation operators for HIFLTSs, aiming to mitigate the uncertainties of decision makers (DMs). A representative numerical example is employed to illustrate the strength and logical soundness of our proposed method in identifying the optimal choice under the given limitations. A comparison examination with the existing TOPSIS approach is also performed to ensure that the results generated with EDAS are accurate.

## Abbreviations

**EDAS**: Evaluation based on distance from average solution
**HIFLTS**: Hesitant intuitionistic fuzzy linguistic term set
**HIFLEWA**: Hesitant intuitionistic fuzzy linguistic Einstein weighted averaging
**DMs**: decision makers
**MCGDM**: Multi-criteria group decision making
**HFS**: Hesitant fuzzy set
**HFLTS**: Hesitant fuzzy linguistic term set

## 1 Introduction

When considering fuzzy decision-making processes and models, MCDM techniques have a wide range of uses. When making essential decisions, decision makers (DMs) can provide profitable insights to help choose from a variety of alternatives. The MCDM process has no limit to the number of criteria that can be taken into consideration; the key factor is how the various alternatives are combined and assessed. Therefore, many researchers have made significant progress in this field using MCDM. Group decision making that takes into account multiple criteria is a beneficial approach to addressing human issues and everyday problems [51]. Complex problems can be addressed with a wide range of experience and fundamental decision-making skills [45]. Among divergent criteria, decision makers can exhibit varied thought processes, understanding of multiple options, and distinct viewpoints. In this context, it is challenging to achieve consistent results from a limited set of potential values.

Fuzzy sets [52] are widely recognized in multiple disciplines for their ability to handle uncertainty and ambiguity. Researchers have provided substantial advances and expansions in fuzzy set theory, resulting in the creation of numerous specialized models. Some generalizations such as Hesitant Fuzzy Sets [38] and Intuitionistic Fuzzy Sets [6] are considered classic, however there are also recent advantages in this domain, such as (2, 1)-fuzzy sets, which can be successfully used in MCDA [2]. Another notable contribution is a generalization of fuzzy soft sets known as (a, b)-fuzzy soft sets, further expanding the scope of fuzzy set theory [4]. Moreover, in 2023, Al-shami and Mhemdi introduced a comprehensive framework for orthopair fuzzy sets, referred to as (m, n)-fuzzy sets, illustrating their utility in various multi-criteria decision-making scenarios [3]. These advances underscore the versatility and applicability of fuzzy sets in addressing complex problems involving uncertainty [30].

Torra [38] suggested the idea of hesitant fuzzy set (HFS) for such outcomes, which has a significant effect in overcoming human reluctance throughout the decision-making process. In the hesitant fuzzy set (HFS) approach [38], several membership values are assigned to various alternatives when encountering conflicting criteria. Rodriguez et al. [34] proposed the concept of hesitant fuzzy linguistic term sets (HFLTSs), effectively addressing fuzziness, thereby inspiring Torra's work on hesitant fuzzy sets (HFS) [38]. Beg and Rashid [7] presented an extension of fuzzy TOPSIS for HFLTS to address MCDM issues. Rodriguez et al. [34] proposed a ranking approach for HFLTS that uses interval values and indices of their envelopes. It is possible that some elements are shared by at least two HFLTSs; however, there is no reason to conclude which HFLTS is superior to the others based on the comparison findings obtained by this method. Wei et al. [43] proposed aggregation theory and comparison approaches for HFLTSs to overcome such problems. For HFLTSs, Wang et al. [40] created dominance relations and constructed directional Hausdorff distance. Dong et al. [12] developed distance measurements for two HFLTSs and proposed a consensus level in linguistic group decision-making problems.

It is not unusual for people to be hesitant when deciding on language terms during the evaluation process. Beg and Rashid [8] have proposed a strategy to address this problem. They introduced the hesitant intuitionistic fuzzy linguistic term set (HIFLTS), which correctly deals with hesitancy. The fuzzy TOPSIS is also a useful supplement for DMs when it comes to HIFLTS regarding alternate criteria. In recent years, the number of research into MCDM problems has increased for HIFLTS as opposed to HFLTS. For example, Liu et al. [27] developed the hesitant intuitionistic fuzzy linguistic set which allows for the possible membership and nonmembership degrees of a component to a linguistic word. Zhou et al. [56] introduced an alternative conceptualization of HIFLTS, termed as intuitionistic hesitant set, and detailed the associated operations for intuitionistic hesitant linguistic numbers. Furthermore, they formulated several aggregation operators and applied them to address MCDM issues. Beg and Rashid [8] along with Faizi et al. [13] developed an outranking method to address the challenges of MCGDM using HIFLTS. Faizi et al. [14] developed metrics for evaluating distances in HIFLTS, incorporating a risk parameter. Rashid et al. [33] propose an ELECTRE-based outranking approach for HIFLTSs, which is also a useful addition to properly handling MCGDM problems. Mahboob et al. [29] developed a method to identify the optimal ideal solution for organizations using hesitant intuitionistic linguistic distribution in collective decision-making scenarios. Liu and Peng [28] unveiled a novel scoring and accuracy function for HIFLTSs, tackling existing limitations and introduced a MULTIMOORA strategy within the HIFLTS framework.

Aggregation operations are essential when dealing with linguistic data in decision-making problems. The operations in dealing with HFS aggregation operators are essentially algebraic operations. In the context of HFS, Xia et al. [44] introduced several aggregation operators and applied them to address MCDM issues based on specific situations. Guo et al. [42] developed the hesitant fuzzy Einstein weighted geometric operator and the hesitant fuzzy Einstein hybrid geometric operator for HFS. Several aggregation operators appropriately handle HFLTS in decision-making challenges, such as Xu [46], [48]. Eienstein aggregation operators [17] have made a significant contribution to contemporary research such as Eienstein t-norm and t-conorm operators. The Eienstein t-norm and t-conorm operators are useful in dealing with ambiguous situations in decision-making problems. Eienstein aggregating operators for hesitant intuitionistic sets were developed by Wang and Liu [41] to address MCGDM problems. Zhao et al. [55] presented the intuitionistic fuzzy Einstein hybrid average and intuitionistic fuzzy Einstein hybrid geometric operators. Kamacı et al. [23] developed dynamic interval-valued picture hesitant fuzzy aggregation Einstein operators to compile the interval-valued picture hesitant fuzzy data gathered over various time frames.

To overcome DMs' hesitancy, there is an anomaly among researchers in planning major new MCDM methods, which has now become a perfect case study. With the help of effective MCDM procedures, difficult situations can be handled with the right choice. Numerous MCDM methods have been established in fuzzy set theory and its different extensions, for example, VIKOR [31], AHP [11], and ANP [21], COPRAS, [39], SAW [20], TOPSIS [9], PROMETHEE [10], TODIM [22] etc. are few MCDM approaches, and more others can be used depending on the situation of the MCDM problem. Keshavarz et al. [18] proposed the EDAS technique for ordinary fuzzy sets for the first time in 2015. This method was later demonstrated to be beneficial and various extensions for ordinary fuzzy sets were created. Stanujkic et al. [36] devised the EDAS approach to deal with Interval Grey Numbers in this manner. This approach was studied by Ghorabaee et al. [19] in particular for interval type-2 fuzzy sets. For hesitant fuzzy linguistic challenges of MCDM, Feng et al. [16] introduced the EDAS approach. Akram et al. [1] developed a comprehensive MAGDM framework that incorporates the significance of the criteria using the intercriteria correlation technique along with the EDAS method. Paul et al. [32] used the cubic pythagorean fuzzy number to encapsulate the fuzzy attributes of the criteria values in relation to the five options within the innovative MCGDM strategy and the cubic pythagorean fuzzy EDAS method. The EDAS approach is widely applicable in everyday scenarios, such as selecting green suppliers selections [54], prioritizing social responsibility projects [24], selecting a carpenter manufacturer [37], applying to hospital selection [25], evaluation of autonomous vehicles [53], and a variety of other topics.

By taking into account the above motivations, our first main objective of this study is to create some useful Einstein operational laws and an Einstein aggregate weighted operator for HIFLTSs that may be utilized to deal with DMs's inclinations in order to tackle real MCGDM situations. Second, we have extended the EDAS approach for HIFLTS in this work by emphasizing the importance of the EDAS method in the current circumstances, which is the main idea of this paper. The innovative contributions of this paper are summarized as follows:

1. We propose several Einstein operational laws for HIFLTSs, accompanied by significant properties and their requisite proofs.
2. To combine uncertain intuitionistic fuzzy linguistic data, the Einstein aggregate weighted operator is developed and utilized to deal with DMs's inclinations in order to tackle real MCGDM situations.
3. In this work, we extended the EDAS approach for HIFLTS by emphasizing the importance of the EDAS method in the current circumstances.
4. The practicality and justification of our suggested approach for choosing the best alternative within the limits are demonstrated using a numerical problem from real life.
5. To confirm that the results generated using EDAS are accurate, a comparison with the existing TOPSIS technique is made.

The remaining portion of this work is organized into the subsequent sections: necessary concepts such as the set of linguistic terms, HFS, HFLTS, and HIFLTS are briefly examined in Section 2. In Section 3, the novel Einstein operational laws for HIFLTSs are defined first, followed by the proof of some key features, including the HIFLEWA operator. The EDAS approach for the MCGDM problem for HIFLTSs is proposed in Section 4. In Section 5, the proposed method is used to solve an existing numerical model. Section 6 provides the conclusion and final observations of the paper.

## 2 Preliminaries

This section investigates fuzzy set, intuitionistic fuzzy set, HFS, linguistic term set, HFLTS, and HIFLTS, as well as their main ideas and actions. We recall the most important definitions from the source works, for the convenience of the reader (cited sources are indicated after each definition).

Definition 2.1 [52] A set *A* in universe *X* associated with a mapping from *X* to [0,1] is called a fuzzy set, symbolized as A={(x,A(x))}. The output A(x) for all x∈X is known as the degree to which *x* belongs to *A* i.e., A(x)=Degree(x∈A) under the membership function A:X→[0,1].

Definition 2.2 [5], [6] An IFS $\overset{˜}{A}$ in *X* is given by $\overset{˜}{A}=\{(x,\mu_{\overset{˜}{A}}(x),\nu_{\overset{˜}{A}}(x))|x\in X\}$ where $\mu_{\overset{˜}{A}}:X\to [0,1]$ and $\nu_{\overset{˜}{A}}:X\to [0,1]$ with the condition that $0\leq \mu_{\overset{˜}{A}}+\nu_{\overset{˜}{A}}\leq 1$ for every x∈X. The numbers $\mu_{\overset{˜}{A}}(x)$, $\nu_{\overset{˜}{A}}(x)\in [0,1]$ denote respectively the degree of membership and non-membership of the element x∈X to the set $\overset{˜}{A}$.

Definition 2.3 [38] Let *X* be a fixed set; an HFS *A* on *X* is defined in terms of a function $h_{A}(x)$, which returns a finite subset of [0,1] when applied to *X*. Mathematically, HFS was expressed by Xia and Xu [44] in the following way:

$$E=\left\{\langle x,h_{E}(x)\rangle |x\;\epsilon X\right\},$$

where $h_{E}(x)$ is a collection of numbers in the range [0,1], signifying the degrees of membership of the element xϵX to the set *E*.

Definition 2.4 [34] Let $S=\left\{s_{t}|t=0,...,g\right\}$ be a finite linguistic term set of odd cardinality, with each $s_{t}$ (0≤t≤g) representing a possible linguistic variable value. The following are the properties of this linguistic term set:

For any $s_{\alpha },s_{\beta }\in S$,

1. neg$(s_{\alpha })=s_{\beta }$, where α+β=g;
2. If $s_{\alpha }\leq s_{\beta }\Leftrightarrow \alpha \leq \beta$, then:
  (a) $max(s_{\alpha },s_{\beta })=s_{\alpha }$, if $s_{\beta }\leq s_{\alpha }$;
  (b) $min(s_{\alpha },s_{\beta })=s_{\alpha }$, if $s_{\alpha }\leq s_{\beta }$.

The operational laws in [46] were described as follows for any two linguistic terms $s_{\alpha },s_{\beta }\in S$ for $\lambda ,\lambda_{1},\lambda_{2}\in [0,1]$.

1. $s_{\alpha }⊕\;s_{\beta }=s_{\beta }⊕\;s_{\alpha }=s_{\alpha +\beta }$;
2. $s_{\alpha }⊗\;s_{\beta }=s_{\beta }⊗\;s_{\alpha }=s_{\alpha \beta }$;
3. $\lambda s_{\alpha }=s_{\lambda \alpha }$;
4. $s_{\alpha }^{\lambda }=s_{\alpha^{\lambda }}$;
5. $\left(\lambda_{1}+\lambda_{2}\right)\;s_{\alpha }=\lambda_{1}s_{\alpha }+\lambda_{2}s_{\alpha }$;
6. $\lambda (s_{\alpha }+\;s_{\beta })=\lambda s_{\alpha }+\lambda s_{\beta }$.

Xu [47], [49] extended discrete linguistic term set *S* to a continuous linguistic term set $\overset{\_}{S}$, such that $\overset{\_}{S}=\left\{s_{t}|0\leq t\leq g\right\}$. If $s_{t}\in S$, the linguistic variable $s_{t}$ is referred to as an original linguistic variable, and if $s_{t}\notin S$, it is referred to as a virtual linguistic variable. During the assessment process, the DM directly provides the original language variable, while the virtual linguistic variable is only employed during the computing process.

Definition 2.5 [35] Let *S* be linguistic term set. A HFLTS, $H_{s}$, is an ordered finite subset of the set of consecutive linguistic terms of *S*.

Liao et al. [26] further refined the definition of HFLTS as follows:

Definition 2.6 [26] Let $x_{i}\epsilon X\;(i=1,2,...,N)$ be fixed and *S* be a linguistic term set. A HFLTS $H_{s}$ on *X* is in mathematical terms can be defined as:

$$H_{s}=\left\{\langle x_{i},h_{s}\left(x_{i}\right)\rangle |x_{i}\epsilon X\right\},$$

where $h_{s}\left(x_{i}\right)$ is the set of possible degrees of linguistic variable $x_{i}$ to the linguistic term set *S* that can be written as:

$$h_{s}\left(x_{i}\right)=\left\{s_{\phi_{l}}\left(x_{i}\right)|s_{\phi_{l}}\epsilon S,\;l=1,2,...L\right\},$$

where *L* is the number of linguistic terms in $h_{s}\left(x_{i}\right)$.

Definition 2.7 [8]: A HIFLTS on *X* are m(x) and n(x) functions that, when applied to *X*, return an ordered finite subset of the set of successive linguistic terms of $S=\left\{s_{\alpha }|\alpha =0,1,...,g\right\}$, which can be represented mathematically as the following:

$$I_{S}=\left\{\langle x,m_{S}(x),n_{S}(x)\rangle |x\;\epsilon X\right\},$$

where $m_{S}(x)$ and $n_{S}(x)$ are subsets of the consecutive linguistic term sets, representing the alternative membership and non-membership degrees of the element xϵX to the set $I_{S}$, with the condition that

$$s_{0}\leq \max(m_{S}(x))⊕\min(n_{S}(x))\leq s_{g}\text{ and}\;s_{0}\leq \min(m_{S}(x))⊕\max(n_{S}(x))\leq s_{g}.$$

We adopt the notation $I_{S}=\left\{m_{S},n_{S}\right\}$ for hesitant intuitionistic fuzzy linguistic element (HIFLE) for clarity.

The following formula can be used to compute the distance between two HIFLTSs:

Definition 2.8 Let $I_{S}^{1}=\left\{m_{S}^{1},n_{S}^{1}\right\}$ and $I_{S}^{2}=\left\{m_{S}^{2},n_{S}^{2}\right\}$ be two HIFLEs where $m_{S}^{1}=\{s_{a_{1}}|s_{a_{1}}\epsilon S,a_{1}=1,2,...\neq m_{S}^{1}\}$, $n_{S}^{1}=\{s_{b_{1}}|s_{b_{1}}\epsilon S,b_{1}=1,2,...\neq n_{S}^{1}\}$, $m_{S}^{2}=\{s_{a_{2}}|s_{a_{2}}\epsilon S,a_{2}=1,2,...\neq m_{S}^{2}\}$ and $n_{S}^{2}=\{s_{b_{2}}|s_{b_{2}}\epsilon S,b_{2}=1,2,...\neq n_{S}^{2}\}$ are HIFLEs having linguistic terms in their membership and non-membership functions respectively. Suppose $\neq m_{S}^{1}=\neq m_{S}^{2}$ and $\neq n_{S}^{1}=\neq n_{S}^{2}$, then the hesitant intuitionistic Euclidean distance measure between $I_{S}^{1}$ and $I_{S}^{2}$ can be defined as follows:

(2.1) $$D\left(I_{S}^{1},I_{S}^{2}\right)=\frac{1}{L}\sqrt{\overset{\neq m_{S}^{1}=\neq m_{S}^{2}}{\sum_{a_{1}=a_{2}=1}}{\left(\alpha_{1}-\alpha_{2}\right)}^{2}+\overset{\neq n_{S}^{1}=\neq n_{S}^{2}}{\sum_{b_{1}=b_{2}=1}}{\left(\beta_{1}-\beta_{2}\right)}^{2}}$$

where $L=\;\neq m_{S}^{1}.\neq n_{S}^{1}.\neq S$. We can easily observe that $D\left(I_{S}^{1},I_{S}^{2}\right)$ satisfies the following conditions:

1. $0\leq D\left(I_{S}^{1},I_{S}^{2}\;\right)\leq 1$
2. $D\left(I_{S}^{1},I_{S}^{2}\;\right)=0$ iff $I_{S}^{1}=I_{S}^{2}$;
3. $D\left(I_{S}^{1},I_{S}^{2}\;\right)=D\left(I_{S}^{1},I_{S}^{2}\;\right)$.

## 3 Novel operational laws for hesitant intuitionistic fuzzy linguistic term sets

In a practical MCDM process, an effective aggregation operator must be used to drive the overall preference value of each choice. Many scholars have developed various aggregation operators to carry out this process in MCDM situations. Recently, Faizi et al. [15] has employed HIFLTS to manage MCDM issues involving unclear information. As mentioned above, Eienstein aggregation operators [17], such as the t-norm and t-conorm operators, have significantly influenced modern research. In decision-making difficulties, the Eienstein t-norm and t-conorm operators are helpful in handling ambiguous circumstances. So our first main concern is to investigate the hesitant intuitionistic fuzzy linguistic aggregation operator based on Einstein operational laws and apply it to MCDM situations.

In this section, we define some HIFLE properties and provide essential proofs, before briefly extending the idea of the EDAS approach for HIFLSs to solve MCGDM problems. Specifically for linguistic variables, Gou et al. [50] established logical operational rules based on two equivalent transformation functions f:Sϵ[0,1] and $f^{-1}:[0,1]\epsilon S$, which can be defined as follows:

$$f(s_{\theta })=\frac{\theta }{g}=\gamma \epsilon [0,1]\;\text{and }f^{-1}(\gamma )=s_{g\gamma }\;\forall s_{\theta }\epsilon S$$

These equivalent transformation functions prove useful when calculating the HIFLE boundaries, as stated in Definition 2.7.

Definition 3.1 Let *S* be a linguistic term set and $I_{S}^{1}=\left\{m_{S}^{1},n_{S}^{1}\right\}$ and $I_{S}^{2}=\left\{m_{S}^{2},n_{S}^{2}\right\}$ be two HIFLEs where $m_{S}^{1}=\{s_{a_{1}}|s_{a_{1}}\epsilon S,a_{1}=1,2,...\neq m_{S_{1}}\}$, $n_{S}^{1}=\{s_{b_{1}}|s_{b_{1}}\epsilon S,b_{1}=1,2,...\neq n_{S_{1}}\}$, $m_{S}^{2}=\{s_{a_{2}}|s_{a_{2}}\epsilon S,a_{2}=1,2,...\neq m_{S_{2}}\}$ and $n_{S}^{2}=\{s_{b_{2}}|s_{b_{2}}\epsilon S,b_{2}=1,2,...\neq n_{S_{2}}\}$. Let $I_{S}=\left\{m_{S},n_{S}\right\}$ be another HIFLE where $m_{S}=\{s_{a}|s_{a}\epsilon S,a=1,2,...\neq m_{S}\}$ and $n_{S}=\{s_{b}|s_{b}\epsilon S,b=1,2,...\neq n_{S}\}$. Then, for any positive real number *λ*, we define

1. $I_{S}^{1}⊕\;I_{S}^{2}=\left(f^{-1}\left(\underset{s_{a_{1}}\in m_{S}^{1},s_{a_{2}}\in m_{S}^{2}}{\cup }\left\{\frac{f(s_{a_{1}})+f(s_{a_{2}})\;}{1+f(s_{a_{1}})f(s_{a_{2}})}\right\}\right),f^{-1}\left(\underset{s_{b_{1}}\in n_{S}^{1},s_{b_{2}}\in n_{S}^{2}}{\cup }\left\{\frac{f(s_{b_{1}})f(s_{b_{2}})}{1+(1-f(s_{b_{1}}))\;.(1-\;f(s_{b_{2}}))}\right\}\right)\right)$

2. $I_{S}^{1}⊗\;I_{S}^{2}=\left(f^{-1}\left(\underset{s_{a_{1}}\in m_{S}^{1},s_{a_{2}}\in m_{S}^{2}}{\cup }\left\{\frac{f(s_{a_{1}})f(s_{a_{2}})}{1+(1-f(s_{a_{1}}))\;.(1-\;f(s_{a_{2}}))}\right\}\right),f^{-1}\left(\underset{s_{b_{1}}\in n_{S}^{1},s_{b_{2}}\in n_{S}^{2}}{\cup }\left\{\frac{f(s_{b_{1}})+f(s_{b_{2}})\;}{1+f(s_{b_{1}})f(s_{b_{2}})}\right\}\right)\right)$

3. $\lambda I_{S}=\left(f^{-1}\left(\underset{s_{a}\in m_{S}}{\cup }\left\{\frac{{{\left(1+f(s_{a})\right)}^{\lambda }\;-\;(1-f(s_{a})))}^{\lambda }\;}{{{\left(1+f(s_{a}))\right)}^{\lambda }\;+\;(1-\;f(s_{a})))}^{\lambda }}\right\}\right),f^{-1}\left(\underset{s_{b}\in n_{S}}{\cup }\left\{\frac{2\;{\left(f(s_{b})\right)}^{\lambda }\;}{{\left(2-f(s_{b})\right)}^{\lambda }\;+\;{\left(f(s_{b})\right)}^{\lambda }}\right\}\right)\right)$

4. $I_{S}^{\lambda }=\left(f^{-1}\left(\underset{s_{a}\in m_{S}}{\cup }\left\{\frac{2\;{\left(f(s_{a})\right)}^{\lambda }\;}{{\left(2-f(s_{a})\right)}^{\lambda }\;+\;{\left(f(s_{a})\right)}^{\lambda }}\right\}\right),f^{-1}\left(\underset{s_{b}\in n_{S}}{\cup }\left\{\frac{{\left(1+f(s_{b})\right)}^{\lambda }\;-\;{\left(1-f(s_{b})\right)}^{\lambda }\;}{{\left(1+f(s_{b})\right)}^{\lambda }\;+\;{\left(1-\;f(s_{b})\right)}^{\lambda }}\right\}\right)\right)$

Theorem 3.2 *Let S be a linguistic term set,* $I_{S}=\left\{m_{S},n_{S}\right\}$ *,* $I_{S}^{1}=\left\{m_{S}^{1},n_{S}^{1}\right\}$ *and* $I_{S}^{2}=\left\{m_{S}^{2},n_{S}^{2}\right\}$ *be three HIFLEs. Then, for any positive real numbers* $\lambda ,\lambda_{1},\lambda_{2}≻0$ *, the HIFLEs* $I_{S},I_{S}^{1}$ *and* $I_{S}^{2}$ *satisfy the following properties.*

1. $I_{S}^{1}⊕\;I_{S}^{2}=I_{S}^{2}⊕I_{S}^{1}$
2. $I_{S}^{1}⊗\;I_{S}^{2}=I_{S}^{2}⊗I_{S}^{1}$
3. $\lambda (I_{S}^{1}⊕\;I_{S}^{2})=\lambda I_{S}^{1}⊕\lambda I_{S}^{2}$
4. ${\left(I_{S}^{1}⊗\;I_{S}^{2}\right)}^{\lambda }={\left(I_{S}^{1}\right)}^{\lambda }⊗{\left(I_{S}^{2}\right)}^{\lambda }$
5. $\lambda_{1}I_{S}⊕\lambda_{2}I_{S}=(\lambda_{1}+\lambda_{2})I_{S}$
6. (IS)λ1⊗(IS)λ2=(IS)+λ2λ1

Proof (1) and (2) are obvious.

3. $\lambda I_{S}^{1}⊕\;\lambda I_{S}^{2}=$

$\left(f^{-1}\left(\underset{s_{a_{1}}\in m_{S}^{1}}{\cup }\left\{\frac{{\left(1+f(s_{a_{1}})\right)}^{\lambda }\;-\;{\left(1-f(s_{a_{1}})\right)}^{\lambda }\;}{{\left(1+f(s_{a_{1}})\right)}^{\lambda }\;+\;{\left(1-\;f(s_{a_{1}})\right)}^{\lambda }}\right\}\right),\;f^{-1}\left(\underset{s_{b_{1}}\in n_{S}^{1}}{\cup }\left\{\frac{2\;{\left(f(s_{b_{1}})\right)}^{\lambda }\;}{{\left(2-f(s_{b_{1}})\right)}^{\lambda }\;+\;{\left(f(s_{b_{1}})\right)}^{\lambda }}\right\}\right)\right)⊕$

$\left(f^{-1}\left(\underset{s_{a_{2}}\in m_{S}^{2}}{\cup }\left\{\frac{{\left(1+f(s_{a_{2}})\right)}^{\lambda }\;-\;{\left(1-f(s_{a_{2}})\right)}^{\lambda }\;}{{\left(1+f(s_{a_{2}})\right)}^{\lambda }\;+\;{\left(1-\;f(s_{a_{2}})\right)}^{\lambda }}\right\}\right),f^{-1}\left(\underset{s_{b_{2}}\in n_{S}^{2}}{\cup }\left\{\frac{2\;{\left(f(s_{b_{2}})\right)}^{\lambda }\;}{{\left(2-f(s_{b_{2}})\right)}^{\lambda }\;+\;{\left(f(s_{b_{2}})\right)}^{\lambda }}\right\}\right)\right)$

$=(f^{-1}\left(\underset{s_{a_{1}}\in m_{S}^{1},s_{a_{2}}\in m_{S}^{2}}{\cup }\left\{\frac{\frac{{\left(1+f(s_{a_{1}})\right)}^{\lambda }\;-\;{\left(1-f(s_{a_{1}})\right)}^{\lambda }\;}{{\left(1+f(s_{a_{1}})\right)}^{\lambda }\;+\;{\left(1-\;f(s_{a_{1}})\right)}^{\lambda }}+\frac{{\left(1+f(s_{a_{2}})\right)}^{\lambda }\;-\;{\left(1-f(s_{a_{2}})\right)}^{\lambda }\;}{{\left(1+f(s_{a_{2}})\right)}^{\lambda }\;+\;{\left(1-\;f(s_{a_{2}})\right)}^{\lambda }}\;}{1+\frac{{\left(1+f(s_{a_{1}})\right)}^{\lambda }\;-\;{\left(1-f(s_{a_{1}})\right)}^{\lambda }\;}{{\left(1+f(s_{a_{1}})\right)}^{\lambda }\;+\;{\left(1-\;f(s_{a_{1}})\right)}^{\lambda }}\frac{{\left(1+f(s_{a_{2}})\right)}^{\lambda }\;-\;{\left(1-f(s_{a_{2}})\right)}^{\lambda }\;}{{\left(1+f(s_{a_{2}})\right)}^{\lambda }\;+\;{\left(1-\;f(s_{a_{2}})\right)}^{\lambda }}}\right\}\right),$

$f^{-1}\left(\underset{s_{b_{1}}\in n_{S}^{1},s_{b_{2}}\in n_{S}^{2}}{\cup }\left\{\frac{\frac{2\;{\left(f(s_{b_{1}})\right)}^{\lambda }\;}{{\left(2-f(s_{b_{1}})\right)}^{\lambda }\;+\;{\left(f(s_{b_{1}})\right)}^{\lambda }}\frac{2\;{\left(f(s_{b_{2}})\right)}^{\lambda }\;}{{\left(2-f(s_{b_{2}})\right)}^{\lambda }\;+\;{\left(f(s_{b_{2}})\right)}^{\lambda }}}{1+\left(1-\frac{2\;{\left(f(s_{b_{1}})\right)}^{\lambda }\;}{{\left(2-f(s_{b_{1}})\right)}^{\lambda }\;+\;{\left(f(s_{b_{1}})\right)}^{\lambda }}\right)\;.\left(1-\;\frac{2\;{\left(f(s_{b_{2}})\right)}^{\lambda }\;}{{\left(2-f(s_{b_{2}})\right)}^{\lambda }\;+\;{\left(f(s_{b_{2}})\right)}^{\lambda }}\right)}\right\}\right))$

$=(f^{-1}\left(\underset{s_{a_{1}}\in m_{S}^{1},s_{a_{2}}\in m_{S}^{2}}{\cup }\left\{\frac{\frac{{\left(1+f(s_{a_{1}})\right)}^{\lambda }{\left(1+f(s_{a_{2}})\right)}^{\lambda }\;}{{\left(1+f(s_{a_{1}})f(s_{a_{2}})\right)}^{\lambda }}\;-\;\frac{{\left(1-f(s_{a_{1}})\right)}^{\lambda }{\left(1-f(s_{a_{2}})\right)}^{\lambda }\;}{{\left(1+f(s_{a_{1}})f(s_{a_{2}})\right)}^{\lambda }}\;}{\frac{{\left(1+f(s_{a_{1}})\right)}^{\lambda }{\left(1+f(s_{a_{2}})\right)}^{\lambda }\;}{{\left(1+f(s_{a_{1}})f(s_{a_{2}})\right)}^{\lambda }}\;+\;\;\frac{{\left(1-f(s_{a_{1}})\right)}^{\lambda }{\left(1-f(s_{a_{2}})\right)}^{\lambda }\;}{{\left(1+f(s_{a_{1}})f(s_{a_{2}})\right)}^{\lambda }}}\right\}\right),$

$f^{-1}\left(\underset{s_{b_{1}}\in n_{S}^{1},s_{b_{2}}\in n_{S}^{2}}{\cup }\left\{\frac{2{\left(f(s_{b_{1}})\right)}^{\lambda }{\left(f(s_{b_{2}})\right)}^{\lambda }\;}{{\left(2-f(s_{b_{1}})\right)}^{\lambda }{\left(2-f(s_{b_{2}})\right)}^{\lambda }+{\left(f(s_{b_{1}})\right)}^{\lambda }{\left(f(s_{b_{2}})\right)}^{\lambda }\;}\right\}\right))$

$=(f^{-1}\left(\underset{s_{a_{1}}\in m_{S}^{1},s_{a_{2}}\in m_{S}^{2}}{\cup }\left\{\frac{{\left(\frac{1+f(s_{a_{1}})f(s_{a_{2}})+f(s_{a_{1}})+f(s_{a_{2}})\;}{1+f(s_{a_{1}})f(s_{a_{2}})}\right)}^{\lambda }\;-\;{\left(\frac{1+f(s_{a_{1}})f(s_{a_{2}})-f(s_{a_{1}})-f(s_{a_{2}})\;}{1+f(s_{a_{1}})f(s_{a_{2}})}\right)}^{\lambda }\;}{{\left(\frac{1+f(s_{a_{1}})f(s_{a_{2}})+f(s_{a_{1}})+f(s_{a_{2}})\;}{1+f(s_{a_{1}})f(s_{a_{2}})}\right)}^{\lambda }\;+\;{\left(\;\frac{1+f(s_{a_{1}})f(s_{a_{2}})-f(s_{a_{1}})-f(s_{a_{2}})\;}{1+f(s_{a_{1}})f(s_{a_{2}})}\right)}^{\lambda }}\right\}\right),$

$f^{-1}\left(\underset{s_{b_{1}}\in n_{S}^{1},s_{b_{2}}\in n_{S}^{2}}{\cup }\left\{\frac{2\;{\left(\frac{f(s_{b_{1}})f(s_{b_{2}})}{1+(1-f(s_{b_{1}}))\;.(1-\;f(s_{b_{2}}))}\right)}^{\lambda }\;}{{\left(2-\frac{f(s_{b_{1}})f(s_{b_{2}})}{1+(1-f(s_{b_{1}}))\;.(1-\;f(s_{b_{2}}))}\right)}^{\lambda }\;+\;{\left(\frac{f(s_{b_{1}})f(s_{b_{2}})}{1+(1-f(s_{b_{1}}))\;.(1-\;f(s_{b_{2}}))}\right)}^{\lambda }}\right\}\right))$

$=\left(f^{-1}\left(\underset{s_{a_{1}}\in m_{S}^{1},s_{a_{2}}\in m_{S}^{2}}{\cup }\left\{\frac{{\left(1+\frac{f(s_{a_{1}})+f(s_{a_{2}})\;}{1+f(s_{a_{1}})f(s_{a_{2}})}\right)}^{\lambda }\;-\;{\left(1-\frac{f(s_{a_{1}})+f(s_{a_{2}})\;}{1+f(s_{a_{1}})f(s_{a_{2}})}\right)}^{\lambda }\;}{{\left(1+\frac{f(s_{a_{1}})+f(s_{a_{2}})\;}{1+f(s_{a_{1}})f(s_{a_{2}})}\right)}^{\lambda }\;+\;{\left(1-\;\frac{f(s_{a_{1}})+f(s_{a_{2}})\;}{1+f(s_{a_{1}})f(s_{a_{2}})}\right)}^{\lambda }}\right\}\right),{\left(n_{S}^{1}⊗\;n_{S}^{2}\right)}^{\lambda }\right)$

$=\left(\lambda (m_{S}^{1}⊕\;m_{S}^{2}),{\left(n_{S}^{1}⊗\;n_{S}^{2}\right)}^{\lambda }\right)$

$=\lambda \left(I_{S}^{1}⊕\;I_{S}^{2}\right)$

4. ${\left(I_{S}^{1}\right)}^{\lambda }⊗{\left(I_{S}^{2}\right)}^{\lambda }=$

$(f^{-1}\left(\underset{s_{a_{1}}\in m_{S}^{1}}{\cup }\left\{\frac{2\;{\left(f(s_{a_{1}})\right)}^{\lambda }\;}{{\left(2-f(s_{a_{1}})\right)}^{\lambda }\;+\;{\left(f(s_{a_{1}})\right)}^{\lambda }}\right\}\right)⊗f^{-1}\left(\underset{s_{a_{2}}\in m_{S}^{2}}{\cup }\left\{\frac{2\;{\left(f(s_{a_{2}})\right)}^{\lambda }\;}{{\left(2-f(s_{a_{2}})\right)}^{\lambda }\;+\;{\left(f(s_{a_{2}})\right)}^{\lambda }}\right\}\right),$

$f^{-1}\left(\underset{s_{b_{1}}\in n_{S}^{1}}{\cup }\left\{\frac{{\left(1+f(s_{b_{1}})\right)}^{\lambda }\;-\;{\left(1-f(s_{b_{1}})\right)}^{\lambda }\;}{{\left(1+f(s_{b_{1}})\right)}^{\lambda }\;+\;{\left(1-\;f(s_{b_{1}})\right)}^{\lambda }}\right\}\right)⊕f^{-1}\left(\underset{s_{b_{2}}\in n_{S}^{2}}{\cup }\left\{\frac{{\left(1+f(s_{b_{2}})\right)}^{\lambda }\;-\;{\left(1-f(s_{b_{2}})\right)}^{\lambda }\;}{{\left(1+f(s_{b_{2}})\right)}^{\lambda }\;+\;{\left(1-\;f(s_{b_{2}})\right)}^{\lambda }}\right\}\right))$

$=(f^{-1}\left(\underset{s_{a_{1}}\in m_{S}^{1},s_{a_{2}}\in m_{S}^{2}}{\cup }\left\{\frac{\frac{2\;{\left(f(s_{a_{1}})\right)}^{\lambda }\;}{{\left(2-f(s_{a_{1}})\right)}^{\lambda }\;+\;{\left(f(s_{a_{1}})\right)}^{\lambda }}\frac{2\;{\left(f(s_{a_{2}})\right)}^{\lambda }\;}{{\left(2-f(s_{a_{2}})\right)}^{\lambda }\;+\;{\left(f(s_{a_{2}})\right)}^{\lambda }}}{1+\left(1-\frac{2\;{\left(f(s_{a_{1}})\right)}^{\lambda }\;}{{\left(2-f(s_{a_{1}})\right)}^{\lambda }\;+\;{\left(f(s_{a_{1}})\right)}^{\lambda }}\right)\;.\left(1-\;\frac{2\;{\left(f(s_{a_{2}})\right)}^{\lambda }\;}{{\left(2-f(s_{a_{2}})\right)}^{\lambda }\;+\;{\left(f(s_{a_{2}})\right)}^{\lambda }}\right)}\right\}\right),$

$f^{-1}\left(\underset{s_{b_{1}}\in n_{S}^{1},s_{b_{2}}\in n_{S}^{2}}{\cup }\left\{\frac{\frac{{\left(1+f(s_{b_{1}})\right)}^{\lambda }\;-\;{\left(1-f(s_{b_{1}})\right)}^{\lambda }\;}{{\left(1+f(s_{b_{1}})\right)}^{\lambda }\;+\;{\left(1-\;f(s_{b_{1}})\right)}^{\lambda }}+\frac{{\left(1+f(s_{b_{2}})\right)}^{\lambda }\;-\;{\left(1-f(s_{b_{2}})\right)}^{\lambda }\;}{{\left(1+f(s_{b_{2}})\right)}^{\lambda }\;+\;{\left(1-\;f(s_{b_{2}})\right)}^{\lambda }}\;}{1+\frac{{\left(1+f(s_{b_{1}})\right)}^{\lambda }\;-\;{\left(1-f(s_{b_{1}})\right)}^{\lambda }\;}{{\left(1+f(s_{b_{1}})\right)}^{\lambda }\;+\;{\left(1-\;f(s_{b_{1}})\right)}^{\lambda }}\frac{{\left(1+f(s_{b_{2}})\right)}^{\lambda }\;-\;{\left(1-f(s_{b_{2}})\right)}^{\lambda }\;}{{\left(1+f(s_{b_{2}})\right)}^{\lambda }\;+\;{\left(1-\;f(s_{b_{2}})\right)}^{\lambda }}}\right\}\right))$

$=(f^{-1}\left(\underset{s_{a_{1}}\in m_{S}^{1},s_{a_{2}}\in m_{S}^{2}}{\cup }\left\{\frac{2{\left(f(s_{a_{1}})\right)}^{\lambda }{\left(f(s_{a_{2}})\right)}^{\lambda }\;}{{\left(2-f(s_{a_{1}})\right)}^{\lambda }{\left(2-f(s_{a_{2}})\right)}^{\lambda }+{\left(f(s_{a_{1}})\right)}^{\lambda }{\left(f(s_{a_{2}})\right)}^{\lambda }\;}\right\}\right),$

$f^{-1}\left(\underset{s_{b_{1}}\in n_{S}^{1},s_{b_{2}}\in n_{S}^{2}}{\cup }\left\{\frac{\frac{{\left(1+f(s_{b_{1}})\right)}^{\lambda }{\left(1+f(s_{b_{2}})\right)}^{\lambda }\;}{{\left(1+f(s_{b_{1}})f(s_{b_{2}})\right)}^{\lambda }}\;-\;\frac{{\left(1-f(s_{b_{1}})\right)}^{\lambda }{\left(1-f(s_{b_{2}})\right)}^{\lambda }\;}{{\left(1+f(s_{b_{1}})f(s_{b_{2}})\right)}^{\lambda }}\;}{\frac{{\left(1+f(s_{b_{1}})\right)}^{\lambda }{\left(1+f(s_{b_{2}})\right)}^{\lambda }\;}{{\left(1+f(s_{b_{1}})f(s_{b_{2}})\right)}^{\lambda }}\;+\;\;\frac{{\left(1-f(s_{b_{1}})\right)}^{\lambda }{\left(1-f(s_{b_{2}})\right)}^{\lambda }\;}{{\left(1+f(s_{b_{1}})f(s_{b_{2}})\right)}^{\lambda }}}\right\}\right))$

$=(f^{-1}\left(\underset{s_{a_{1}}\in m_{S}^{1},s_{a_{2}}\in m_{S}^{2}}{\cup }\left\{\frac{2\;{\left(\frac{f(s_{a_{1}})f(s_{a_{2}})}{1+(1-f(s_{a_{1}}))\;.(1-\;f(s_{a_{2}}))}\right)}^{\lambda }\;}{{\left(2-\frac{f(s_{a_{1}})f(s_{a_{2}})}{1+(1-f(s_{a_{1}}))\;.(1-\;f(s_{a_{2}}))}\right)}^{\lambda }\;+\;{\left(\frac{f(s_{a_{1}})f(s_{a_{2}})}{1+(1-f(s_{a_{1}}))\;.(1-\;f(s_{a_{2}}))}\right)}^{\lambda }}\right\}\right),$

$f^{-1}\left(\underset{s_{b_{1}}\in n_{S}^{1},s_{b_{2}}\in n_{S}^{2}}{\cup }\left\{\frac{{\left(\frac{1+f(s_{b_{1}})f(s_{b_{2}})+f(s_{b_{1}})+f(s_{b_{2}})\;}{1+f(s_{b_{1}})f(s_{b_{2}})}\right)}^{\lambda }\;-\;{\left(\frac{1+f(s_{b_{1}})f(s_{b_{2}})-f(s_{b_{1}})-f(s_{b_{2}})\;}{1+f(s_{b_{1}})f(s_{b_{2}})}\right)}^{\lambda }\;}{{\left(\frac{1+f(s_{b_{1}})f(s_{b_{2}})+f(s_{b_{1}})+f(s_{b_{2}})\;}{1+f(s_{b_{1}})f(s_{b_{2}})}\right)}^{\lambda }\;+\;{\left(\;\frac{1+f(s_{b_{1}})f(s_{b_{2}})-f(s_{b_{1}})-f(s_{b_{2}})\;}{1+f(s_{b_{1}})f(s_{b_{2}})}\right)}^{\lambda }}\right\}\right))$

$={\left(m_{S}^{1}⊗\;m_{S}^{2}\right)}^{\lambda },f^{-1}\left(\underset{s_{b_{1}}\in n_{S}^{1},s_{b_{2}}\in n_{S}^{2}}{\cup }\left\{\frac{{\left(1+\frac{f(s_{b_{1}})+f(s_{b_{2}})\;}{1+f(s_{b_{1}})f(s_{b_{2}})}\right)}^{\lambda }\;-\;{\left(1-\frac{f(s_{b_{1}})+f(s_{b_{2}})\;}{1+f(s_{b_{1}})f(s_{b_{2}})}\right)}^{\lambda }\;}{{\left(1+\frac{f(s_{b_{1}})+f(s_{b_{2}})\;}{1+f(s_{b_{1}})f(s_{b_{2}})}\right)}^{\lambda }\;+\;{\left(1-\;\frac{f(s_{b_{1}})+f(s_{b_{2}})\;}{1+f(s_{b_{1}})f(s_{b_{2}})}\right)}^{\lambda }}\right\}\right)$

$={\left(m_{S}^{1}⊗\;m_{S}^{2}\right)}^{\lambda },\lambda \left(n_{S}^{1}⊕n_{S}^{2}\right)$

$={\left(I_{S}^{1}\right)}^{\lambda }⊗{\left(I_{S}^{2}\right)}^{\lambda }$

5. $\lambda_{1}I_{S}⊕\lambda_{2}I_{S}=$

$(f^{-1}\left(\underset{s_{a}\in m_{S}}{\cup }\left\{\frac{{\left(1+f(s_{a})\right)}^{\lambda_{1}}\;-\;{\left(1-f(s_{a})\right)}^{\lambda_{1}}\;}{{\left(1+f(s_{a})\right)}^{\lambda_{1}}\;+\;{\left(1-\;f(s_{a})\right)}^{\lambda_{1}}}\right\}\right)⊕f^{-1}\left(\underset{s_{a}\in m_{S}}{\cup }\left\{\frac{{\left(1+f(s_{a})\right)}^{\lambda_{2}}\;-\;{\left(1-f(s_{a})\right)}^{\lambda_{2}}\;}{{\left(1+f(s_{a})\right)}^{\lambda_{2}}\;+\;{\left(1-\;f(s_{a})\right)}^{\lambda_{2}}}\right\}\right),$

$f^{-1}\left(\underset{s_{b}\in n_{S}}{\cup }\left\{\frac{2\;{\left(f(s_{b})\right)}^{\lambda_{1}}\;}{{\left(2-f(s_{b})\right)}^{\lambda_{1}}\;+\;{\left(f(s_{b})\right)}^{\lambda_{1}}}\right\}\right)⊗f^{-1}\left(\underset{s_{b}\in n_{S}}{\cup }\left\{\frac{2\;{\left(f(s_{b})\right)}^{\lambda_{2}}\;}{{\left(2-f(s_{b})\right)}^{\lambda_{2}}\;+\;{\left(f(s_{b})\right)}^{\lambda_{2}}}\right\}\right))$

$=(f^{-1}\left(\underset{s_{a}\in m_{S}}{\cup }\left\{\frac{\frac{{\left(1+f(s_{a})\right)}^{\lambda_{1}}\;-\;{\left(1-f(s_{a})\right)}^{\lambda_{1}}\;}{{\left(1+f(s_{a})\right)}^{\lambda_{1}}\;+\;{\left(1-\;f(s_{a})\right)}^{\lambda_{1}}}+\frac{{\left(1+f(s_{a})\right)}^{\lambda_{2}}\;-\;{\left(1-f(s_{a})\right)}^{\lambda_{2}}\;}{{\left(1+f(s_{a})\right)}^{\lambda_{2}}\;+\;{\left(1-\;f(s_{a})\right)}^{\lambda_{2}}}\;}{1+\left(\frac{{\left(1+f(s_{a})\right)}^{\lambda_{1}}\;-\;{\left(1-f(s_{a})\right)}^{\lambda_{1}}\;}{{\left(1+f(s_{a})\right)}^{\lambda_{1}}\;+\;{\left(1-\;f(s_{a})\right)}^{\lambda_{1}}}\right)\left(\frac{{\left(1+f(s_{a})\right)}^{\lambda_{2}}\;-\;{\left(1-f(s_{a})\right)}^{\lambda_{2}}\;}{{\left(1+f(s_{a})\right)}^{\lambda_{2}}\;+\;{\left(1-\;f(s_{a})\right)}^{\lambda_{2}}}\right)}\right\}\right),$

$f^{-1}\left(\underset{s_{b}\in n_{S}}{\cup }\left\{\frac{\left(\frac{2\;{\left(f(s_{b})\right)}^{\lambda_{1}}\;}{{\left(2-f(s_{b})\right)}^{\lambda_{1}}\;+\;{\left(f(s_{b})\right)}^{\lambda_{1}}}\right)\left(\frac{2\;{\left(f(s_{b})\right)}^{\lambda_{2}}\;}{{\left(2-f(s_{b})\right)}^{\lambda_{2}}\;+\;{\left(f(s_{b})\right)}^{\lambda_{2}}}\right)}{1+\left(1-\frac{2\;{\left(f(s_{b})\right)}^{\lambda_{1}}\;}{{\left(2-f(s_{b})\right)}^{\lambda_{1}}\;+\;{\left(f(s_{b})\right)}^{\lambda_{1}}}\right)\;.\left(1-\;\frac{2\;{\left(f(s_{b})\right)}^{\lambda_{2}}\;}{{\left(2-f(s_{b})\right)}^{\lambda_{2}}\;+\;{\left(f(s_{b})\right)}^{\lambda_{2}}}\right)}\right\}\right))$

$=f^{-1}\left(\underset{s_{a}\in m_{S}}{\cup }\left\{\frac{{\left(1+f(s_{a})\right)}^{\lambda_{1}+\lambda_{2}}-\;{\left(1-f(s_{a})\right)}^{\lambda_{1}+\lambda_{2}}\;}{\;{\left(1+f(s_{a})\right)}^{\lambda_{1}+\lambda_{2}}+{\left(1-f(s_{a})\right)}^{\lambda_{1}+\lambda_{2}}}\right\}\right),f^{-1}\left(\underset{s_{b}\in n_{S}}{\cup }\left\{\frac{2{\left(f(s_{b})\right)}^{\lambda_{1}+\lambda_{2}}\;}{{\left(2-f(s_{b})\right)}^{\lambda_{1}+\lambda_{2}}\;+{\left(f(s_{b})\right)}^{\lambda_{1}+\lambda_{2}}}\right\}\right)$

$=\left\{\left(\lambda_{1}+\lambda_{2}\right)m_{S},{\left(n_{S}\right)}^{\lambda_{1}+\lambda_{2}}\right\}$

$=(\lambda_{1}+\lambda_{2})I_{S}$

6. ${\left(I_{S}\right)}^{\lambda_{1}}⊗{\left(I_{S}\right)}^{\lambda_{2}}=$

$(f^{-1}\left(\underset{s_{a}\in m_{S}}{\cup }\left\{\frac{2\;{\left(f(s_{a})\right)}^{\lambda_{1}}\;}{{\left(2-f(s_{a})\right)}^{\lambda_{1}}\;+\;{\left(f(s_{a})\right)}^{\lambda_{1}}}\right\}\right)⊗f^{-1}\left(\underset{s_{a}\in m_{S}}{\cup }\left\{\frac{2\;{\left(f(s_{a})\right)}^{\lambda_{2}}\;}{{\left(2-f(s_{a})\right)}^{\lambda_{2}}\;+\;{\left(f(s_{a})\right)}^{\lambda_{2}}}\right\}\right),$

$f^{-1}\left(\underset{s_{b}\in n_{S}}{\cup }\left\{\frac{{\left(1+f(s_{b})\right)}^{\lambda_{1}}\;-\;{\left(1-f(s_{b})\right)}^{\lambda_{1}}\;}{{\left(1+f(s_{b})\right)}^{\lambda_{1}}\;+\;{\left(1-\;f(s_{b})\right)}^{\lambda_{1}}}\right\}\right)⊕f^{-1}\left(\underset{s_{b}\in n_{S}}{\cup }\left\{\frac{{\left(1+f(s_{b})\right)}^{\lambda_{2}}\;-\;{\left(1-f(s_{b})\right)}^{\lambda_{2}}\;}{{\left(1+f(s_{b})\right)}^{\lambda_{2}}\;+\;{\left(1-\;f(s_{b})\right)}^{\lambda_{2}}}\right\}\right))$

$=(f^{-1}\left(\underset{s_{a}\in m_{S}}{\cup }\left\{\frac{\left(\frac{2\;{\left(f(s_{a})\right)}^{\lambda_{1}}\;}{{\left(2-f(s_{a})\right)}^{\lambda_{1}}\;+\;{\left(f(s_{a})\right)}^{\lambda_{1}}}\right)\left(\frac{2\;{\left(f(s_{a})\right)}^{\lambda_{2}}\;}{{\left(2-f(s_{a})\right)}^{\lambda_{2}}\;+\;{\left(f(s_{a})\right)}^{\lambda_{2}}}\right)}{1+\left(1-\frac{2\;{\left(f(s_{a})\right)}^{\lambda_{1}}\;}{{\left(2-f(s_{a})\right)}^{\lambda_{1}}\;+\;{\left(f(s_{a})\right)}^{\lambda_{1}}}\right)\;.\left(1-\;\frac{2\;{\left(f(s_{a})\right)}^{\lambda_{2}}\;}{{\left(2-f(s_{a})\right)}^{\lambda_{2}}\;+\;{\left(f(s_{a})\right)}^{\lambda_{2}}}\right)}\right\}\right),$

$f^{-1}\left(\underset{s_{b}\in n_{S}}{\cup }\left\{\frac{\frac{{\left(1+f(s_{b})\right)}^{\lambda_{1}}\;-\;{\left(1-f(s_{b})\right)}^{\lambda_{1}}\;}{{\left(1+f(s_{b})\right)}^{\lambda_{1}}\;+\;{\left(1-\;f(s_{b})\right)}^{\lambda_{1}}}+\frac{{\left(1+f(s_{b})\right)}^{\lambda_{2}}\;-\;{\left(1-f(s_{b})\right)}^{\lambda_{2}}\;}{{\left(1+f(s_{b})\right)}^{\lambda_{2}}\;+\;{\left(1-\;f(s_{b})\right)}^{\lambda_{2}}}\;}{1+\left(\frac{{\left(1+f(s_{b})\right)}^{\lambda_{1}}\;-\;{\left(1-f(s_{b})\right)}^{\lambda_{1}}\;}{{\left(1+f(s_{b})\right)}^{\lambda_{1}}\;+\;{\left(1-\;f(s_{b})\right)}^{\lambda_{1}}}\right)\left(\frac{{\left(1+f(s_{b})\right)}^{\lambda_{2}}\;-\;{\left(1-f(s_{b})\right)}^{\lambda_{2}}\;}{{\left(1+f(s_{b})\right)}^{\lambda_{2}}\;+\;{\left(1-\;f(s_{b})\right)}^{\lambda_{2}}}\right)}\right\}\right))$

$=f^{-1}\left(\underset{s_{a}\in m_{S}}{\cup }\left\{\frac{2{\left(f(s_{a})\right)}^{\lambda_{1}+\lambda_{2}}\;}{{\left(2-f(s_{a})\right)}^{\lambda_{1}+\lambda_{2}}\;+{\left(f(s_{a})\right)}^{\lambda_{1}+\lambda_{2}}}\right\}\right),f^{-1}\left(\underset{s_{b}\in n_{S}}{\cup }\left\{\frac{{\left(1+f(s_{b})\right)}^{\lambda_{1}+\lambda_{2}}-\;{\left(1-f(s_{b})\right)}^{\lambda_{1}+\lambda_{2}}\;}{\;{\left(1+f(s_{b})\right)}^{\lambda_{1}+\lambda_{2}}+{\left(1-f(s_{b})\right)}^{\lambda_{1}+\lambda_{2}}}\right\}\right)$

=(mS)+λ2λ1,(λ1+λ2)nS

$={\left(I_{S}\right)}^{\lambda_{1}+\lambda_{2}}$ □

Definition 3.3 Let $I_{S}^{1},I_{S}^{2},...I_{S}^{n}$ be HIFLEs and $w={\left(w_{1},...,w_{n}\right)}^{T}$ be the weight vector of $I_{S}^{j}(j=1,...,n)$ with $w_{j}$ ∈[0,1] such that $\sum_{j=1}^{n}wj=1$. Then, HIFLEWA operator can be defined as

HIFLEWA(IS1,IS2,...ISn)=(f(∪saj∈mSj{∏j=1n(1+f(saj))  wj  −  ∏j=1n(1−f(saj))wj  ∏j=1n(1+f(saj))wj  +  ∏j=1n(1−f(saj))wj})−1,

$f^{-1}\left(\underset{s_{b_{j}}\in n_{S}^{j}}{\cup }\left\{\frac{2\prod_{j=1}^{n}f\;{\left(s_{b_{j}}\right)}^{w_{j}}}{\prod_{j=1}^{n}{\left(2-f\;(s_{b_{j}})\right)}^{w_{j}}+\;\prod_{j=1}^{n}{\left(\;f\;(s_{b_{j}})\right)}^{w_{j}}}\right\}\right))$.

It can be easily verified that $HIFLEWA\left(I_{S}^{1},I_{S}^{2},...I_{S}^{n}\right)$ is also a HIFLE.

Theorem 3.4 *Let* $I_{S}^{1},I_{S}^{2},...I_{S}^{n}$ *be* HIFLEs *and* $w={\left(w_{1},...,w_{n}\right)}^{T}$ *be the weight vector of* $I_{S}^{j}(j=1,...,n)$ *with* $w_{j}$ ∈[0,1] *such that* $\sum_{j=1}^{n}w_{j}=1$ *; then the following properties hold.*

1. **Idempotency:** If $I_{S}^{1}=...=I_{S}^{n}=I_{S}$, then $HIFLEWA\left(I_{S}^{1},I_{S}^{2},...I_{S}^{n}\right)=I_{S}$.
2. **Boundary:** If $\underset{j=1}{\overset{n}{\min}}\left(I_{S}^{j}\right)=I_{s_{\min}}=\left(m_{s_{\min}},n_{s_{\max}}\right)$ and $\underset{j=1}{\overset{n}{\max}}\left(I_{S}^{j}\right)=I_{s_{\max}}=\left(m_{s_{\max}},n_{s_{\min}}\right)$, such that $m_{s_{\min}}\leq m_{s_{i}}$, $n_{s_{\max}}\geq n_{s_{i}}$ and $m_{s_{\max}}\geq m_{s_{i}}$, $n_{s_{\min}}\leq n_{s_{i}}$ for *i*, then $I_{s_{\min}}\leq HIFLEWA(I_{S}^{1},...,I_{S}^{n})\leq I_{s_{\max}}$.
3 **Monotonicity:** Let $\left\{I_{S}^{⁎}=\left(m_{s_{j}}^{⁎},n_{s_{j}}^{⁎}\right),(j=1,2,...,n)\right\}$ be a set of HIFLEs such that $m_{s_{j}}^{⁎}\geq n_{s_{i}}$ and $n_{s_{j}}^{⁎}\leq n_{s_{i}}$ for any *i*, then $HIFLEWA(I_{S}^{⁎},...,I_{S}^{⁎})\geq HIFLEWA(I_{S}^{1},...,I_{S}^{n})$ for any *w*.

Proof 1. **Idempotency:**

HIFLEWA(IS1,IS2,...ISn)=(f(∪sa∈mS{∏j=1n(1+f(sa))  wj  −  ∏j=1n(1−f(sa))wj  ∏j=1n(1+f(sa))wj  +  ∏j=1n(1−f(sa))wj})−1,

$f^{-1}\left(\underset{\;s_{b}\in n_{S}}{\cup }\left\{\frac{2\prod_{j=1}^{n}\;{\left(f(s_{b})\right)}^{w_{j}}}{\prod_{j=1}^{n}{\left(2-f(s_{b})\right)}^{w_{j}}+\;\prod_{j=1}^{n}{\left(\;f(s_{b})\right)}^{w_{j}}}\right\}\right))$

=(f(∪sa∈mS{(1+f(sa))  −  (1−f(sa))  (1+f(sa))  +  (1−f(sa))})−1,f−1(∪sb∈nS{2f(sb)(2−f(sb))+f(sb)}))

=(f(∪sa∈mS{  f(sa)})−1,f−1(∪sb∈nS{f(sb)}))

$=\left(m_{S},n_{S}\right)=I_{S}$

2. **Boundary:**

Let $J_{1}(y_{1})=\frac{1-y_{1}}{1+y_{1}},\;y_{1}\in [0,1]$, then $J_{1}^{\prime }(y_{1})=\frac{-2}{{\left(1+y_{1}\right)}^{2}}<0$. So, $J_{1}(y_{1})$ is a decreasing function. Since $f(s_{a\min})\leq f(s_{a_{j}})\leq f(s_{a\max})$, for all *j*, then $J_{1}\left(f(s_{a\max})\right)\leq J_{1}(f(s_{a_{j}}))\leq J_{1}\left(f(s_{a\min})\right)$, for all *j*. Therefore for j=1,2,...,n,

$\underset{s_{a\max}\in m_{s_{\max}}}{\cup }\left\{\frac{1-f(s_{a\max})}{1+f(s_{a\max})}\right\}\leq \underset{s_{a_{j}}\in m_{S}^{j}}{\cup }\left\{\frac{1-f(s_{a_{j}})}{1+f(s_{a_{j}})}\right\}\leq \underset{s_{a_{\min}}\in m_{s_{\min}}}{\cup }\left\{\frac{1-f(s_{a_{\min}})}{1+f(s_{a_{\min}})}\right\}$,

$\underset{s_{a\max}\in m_{s_{\max}}}{\cup }\left\{\prod_{j=1}^{n}{\left(\frac{1-f(s_{a\max})}{1+f(s_{a\max})}\right)}^{w_{j}}\right\}\leq \underset{s_{a_{j}}\in m_{S}^{j}}{\cup }\left\{\prod_{j=1}^{n}{\left(\frac{1-f(s_{a_{j}})}{1+f(s_{a_{j}})}\right)}^{w_{j}}\right\}$

$\leq \underset{s_{a_{\min}}\in m_{s_{\min}}}{\cup }\left\{\prod_{j=1}^{n}{\left(\frac{1-f(s_{a_{\min}})}{1+f(s_{a_{\min}})}\right)}^{w_{j}}\right\}$

$\underset{s_{a\max}\in m_{s_{\max}}}{\cup }\left\{{\left(\frac{1-f(s_{a\max})}{1+f(s_{a\max})}\right)}^{\sum_{j=1}^{n}w_{j}}\right\}\leq \underset{s_{a_{j}}\in m_{S}^{j}}{\cup }\left\{\prod_{j=1}^{n}{\left(\frac{1-f(s_{a_{j}})}{1+f(s_{a_{j}})}\right)}^{w_{j}}\right\}$

$\leq \underset{s_{a_{\min}}\in m_{s_{\min}}}{\cup }\left\{{\left(\frac{1-f(s_{a_{\min}})}{1+f(s_{a_{\min}})}\right)}^{\sum_{j=1}^{n}w_{j}}\right\}$

$\underset{s_{a\max}\in m_{s_{\max}}}{\cup }\left\{\left(\frac{1-f(s_{a\max})}{1+f(s_{a\max})}\right)\right\}\leq \underset{s_{a_{j}}\in m_{S}^{j}}{\cup }\left\{\prod_{j=1}^{n}{\left(\frac{1-f(s_{a_{j}})}{1+f(s_{a_{j}})}\right)}^{w_{j}}\right\}$

$\leq \underset{s_{a_{\min}}\in m_{s_{\min}}}{\cup }\left\{\left(\frac{1-f(s_{a_{\min}})}{1+f(s_{a_{\min}})}\right)\right\}$

$\underset{s_{a\max}\in m_{s_{\max}}}{\cup }\left\{\frac{2}{1+f(s_{a\max})}\right\}\leq \underset{s_{a_{j}}\in m_{S}^{j}}{\cup }\left\{\prod_{j=1}^{n}{\left(\frac{1-f(s_{a_{j}})}{1+f(s_{a_{j}})}\right)}^{w_{j}}+1\right\}\leq \underset{s_{a_{\min}}\in m_{s_{\min}}}{\cup }\left\{\frac{2}{1+f(s_{a_{\min}})}\right\}$

$\underset{s_{a_{\min}}\in m_{s_{\min}}}{\cup }\left\{\frac{1+f(s_{a_{\min}})}{2}\right\}\leq \underset{s_{a_{j}}\in m_{S}^{j}}{\cup }\left\{\frac{1}{\prod_{j=1}^{n}{\left(\frac{1-f(s_{a_{j}})}{1+f(s_{a_{j}})}\right)}^{w_{j}}+1}\right\}\leq \underset{s_{a\max}\in m_{s_{\max}}}{\cup }\left\{\frac{1+f(s_{a\max})}{2}\right\}$

$\underset{s_{a_{\min}}\in m_{s_{\min}}}{\cup }\left\{1+f(s_{a_{\min}})\right\}\leq \underset{s_{a_{j}}\in m_{S}^{j}}{\cup }\left\{\frac{2\prod_{j=1}^{n}{\left(1+f(s_{a_{j}})\right)}^{w_{j}}}{\prod_{j=1}^{n}{\left(1-f(s_{a_{j}})\right)}^{w_{j}}+\prod_{j=1}^{n}{\left(1+f(s_{a_{j}})\right)}^{w_{j}}}\right\}$

$\leq \underset{s_{a\max}\in m_{s_{\max}}}{\cup }\left\{1+f(s_{a\max})\right\}$

$f^{-1}\left(\underset{s_{a_{\min}}\in m_{s_{\min}}}{\cup }\left\{f(s_{a_{\min}})\right\}\right)\leq f^{-1}\left(\underset{s_{a_{j}}\in m_{S}^{j}}{\cup }\left\{\frac{\prod_{j=1}^{n}{\left(1+f(s_{a_{j}})\right)}^{wj}-\prod_{j=1}^{n}{\left(1-f(s_{a_{j}})\right)}^{wj}}{\prod_{j=1}^{n}{\left(1+f(s_{a_{j}})\right)}^{w_{j}}+\prod_{j=1}^{n}{\left(1-f(s_{a_{j}})\right)}^{w_{j}}}\right\}\right)$

$\leq f^{-1}\left(\underset{s_{a\max}\in m_{s_{\max}}}{\cup }\left\{f(s_{a\max})\right\}\right)$

(3.1) $$m_{s_{\min}}\leq f^{-1}\left(\underset{s_{a_{j}}\in m_{S}^{j}}{\cup }\left\{\frac{\prod_{j=1}^{n}{\left(1+f(s_{a_{j}})\right)}^{w_{j}}-\prod_{j=1}^{n}{\left(1-f(s_{a_{j}})\right)}^{w_{j}}}{\prod_{j=1}^{n}{\left(1+f(s_{a_{j}})\right)}^{w_{j}}+\prod_{j=1}^{n}{\left(1-f(s_{a_{j}})\right)}^{w_{j}}}\right\}\right)\leq m_{s_{\max}}$$

Let $h(x_{1})=\frac{2-x_{1}}{x_{1}},x_{1}\in (0,1]$, then $h^{\prime }(x_{1})=\frac{-2}{x_{1}^{2}}<0$ which shows $h(x_{1})$ is decreasing function on (0,1]. So if $f(s_{b_{\max}})\leq f(s_{b_{j}})\leq f(s_{b_{\min}})$, for all *j*, then $h(f(s_{b_{\min}}))\leq h(f(s_{b_{j}}))\leq h(f(s_{b_{\max}}))$, j=1,2,...,n. Therefore,

$\underset{s_{b_{\min}}\in n_{s_{\min}}}{\cup }\left\{\frac{2-f(s_{b_{\min}})}{f(s_{b_{\min}})}\right\}\leq \underset{s_{b_{j}}\in n_{S}^{j}}{\cup }\left\{\frac{2-f(s_{b_{j}})}{f(s_{b_{j}})}\right\}\leq \underset{s_{b_{\max}}\in n_{s_{\max}}}{\cup }\left\{\frac{2-f(s_{b_{\max}})}{f(s_{b_{\max}})}\right\}$

$\underset{s_{b_{\min}}\in n_{s_{\min}}}{\cup }\left\{\prod_{j=1}^{n}{\left(\frac{2-f(s_{b_{\min}})}{f(s_{b_{\min}})}\right)}^{w_{j}}\right\}\leq \underset{s_{b_{j}}\in n_{S}^{j}}{\cup }\left\{\prod_{j=1}^{n}{\left(\frac{2-f(s_{b_{j}})}{f(s_{b_{j}})}\right)}^{w_{j}}\right\}$

$\leq \underset{s_{b_{\max}}\in n_{s_{\max}}}{\cup }\left\{\prod_{j=1}^{n}{\left(\frac{2-f(s_{b_{\max}})}{f(s_{b_{\max}})}\right)}^{w_{j}}\right\}$

$\underset{s_{b_{\min}}\in n_{s_{\min}}}{\cup }\left\{{\left(\frac{2-f(s_{b_{\min}})}{f(s_{b_{\min}})}\right)}^{\sum_{j=1}^{n}w_{j}}\right\}\leq \underset{s_{b_{j}}\in n_{S}^{j}}{\cup }\left\{\prod_{j=1}^{n}{\left(\frac{2-f(s_{b_{j}})}{f(s_{b_{j}})}\right)}^{w_{j}}\right\}$

$\leq \underset{s_{b_{\max}}\in n_{s_{\max}}}{\cup }\left\{{\left(\frac{2-f(s_{b_{\max}})}{f(s_{b_{\max}})}\right)}^{\sum_{j=1}^{n}w_{j}}\right\}$

$\underset{s_{b_{\min}}\in n_{s_{\min}}}{\cup }\left\{\frac{2-f(s_{b_{\min}})}{f(s_{b_{\min}})}\right\}\leq \underset{s_{b_{j}}\in n_{S}^{j}}{\cup }\left\{\prod_{j=1}^{n}{\left(\frac{2-f(s_{b_{j}})}{f(s_{b_{j}})}\right)}^{w_{j}}\right\}\leq \underset{s_{b_{\max}}\in n_{s_{\max}}}{\cup }\left\{\frac{2-f(s_{b_{\max}})}{f(s_{b_{\max}})}\right\}$

$\underset{s_{b_{\min}}\in n_{s_{\min}}}{\cup }\left\{\frac{2}{f(s_{b_{\min}})}\right\}\leq \underset{s_{b_{j}}\in n_{S}^{j}}{\cup }\left\{\prod_{k=1}^{n}{\left(\frac{2-f(s_{b_{j}})}{f(s_{b_{j}})}\right)}^{w_{j}}+1\right\}\leq \underset{s_{b_{\max}}\in n_{s_{\max}}}{\cup }\left\{\frac{2}{f(s_{b_{\max}})}\right\}$

$f^{-1}\left(\underset{s_{b_{\max}}\in n_{s_{\max}}}{\cup }\left\{f(s_{b_{\max}})\right\}\right)\leq f^{-1}\left(\underset{s_{b_{j}}\in n_{S}^{j}}{\cup }\left\{\frac{2{\left(f(s_{b_{j}})\right)}^{w_{j}}}{\prod_{j=1}^{n}{\left(2-f(s_{b_{j}})\right)}^{w_{j}}+\prod_{j=1}^{n}{\left(f(s_{b_{j}})\right)}^{w_{j}}}\right\}\right)$

$\leq f^{-1}\left(\underset{s_{b_{\min}}\in n_{s_{\min}}}{\cup }\left\{f(s_{b_{\min}})\right\}\right)$

(3.2) $$n_{s_{\max}}\leq f^{-1}\left(\underset{s_{b_{j}}\in n_{S}^{j}}{\cup }\left\{\frac{2{\left(f(s_{b_{j}})\right)}^{w_{j}}}{\prod_{j=1}^{n}{\left(2-f(s_{b_{j}})\right)}^{w_{j}}+\prod_{j=1}^{n}{\left(f(s_{b_{j}})\right)}^{w_{j}}}\right\}\right)\leq n_{s_{\min}}$$

Hence $I_{s_{\min}}\leq HIFLEWA(I_{S}^{1},...,I_{S}^{n})\leq I_{s_{\max}}$. by using the inequalities (3.1) and (3.2).

**Monotonicity:**

Let $J_{1}(y_{1})=\frac{1-y_{1}}{1+y_{1}},y_{1}\in [0,1]$, then $J_{1}^{\prime }(y_{1})=\frac{-2}{{\left(1+y_{1}\right)}^{2}}<0$, so, $J_{1}(y_{1})$ is a decreasing function. If $f(s_{a_{j}})\leq f(s_{a_{j}^{⁎}})$, then $J_{1}\left(f(s_{a_{j}})\right)\geq J_{1}\left(f(s_{a_{j}^{⁎}})\right)$, for all *j*. This implies

$\underset{s_{a_{j}}\in m_{S}^{j}}{\cup }\left\{\frac{1-f(s_{a_{j}})}{1+f(s_{a_{j}})}\right\}\geq \underset{s_{a_{j}^{⁎}}\in m_{S}^{j^{⁎}}}{\cup }\left\{\frac{1-f(s_{a_{j}^{⁎}})}{1+f(s_{a_{j}^{⁎}})}\right\},\;j=1,2,...,n$

$\underset{s_{a_{j}}\in m_{S}^{j}}{\cup }\left\{\prod_{j=1}^{n}{\left(\frac{1-f(s_{a_{j}})}{1+f(s_{a_{j}})}\right)}^{w_{j}}\right\}\geq \underset{s_{a_{j}^{⁎}}\in m_{S}^{j^{⁎}}}{\cup }\left\{\prod_{j=1}^{n}{\left(\frac{1-f(s_{a_{j}^{⁎}})}{1+f(s_{a_{j}^{⁎}})}\right)}^{w_{j}}\right\}$

$\underset{s_{a_{j}^{⁎}}\in m_{S}^{j^{⁎}}}{\cup }\left\{\frac{1}{\prod_{j=1}^{n}{\left(\frac{1-f(s_{a_{j}^{⁎}})}{1+f(s_{a_{j}^{⁎}})}\right)}^{w_{j}}+1}\right\}\geq \underset{s_{a_{j}}\in m_{S}^{j}}{\cup }\left\{\frac{1}{\prod_{j=1}^{n}{\left(f(s_{a_{j}})\right)}^{w_{j}}+1}\right\}$

$\underset{s_{a_{j}^{⁎}}\in m_{S}^{j^{⁎}}}{\cup }\left\{\frac{2}{\prod_{j=1}^{n}{\left(\frac{1-f(s_{a_{j}^{⁎}})}{1+f(s_{a_{j}^{⁎}})}\right)}^{w_{j}}+1}\right\}\geq \underset{s_{a_{j}}\in m_{S}^{j}}{\cup }\left\{\frac{2}{\prod_{j=1}^{n}{\left(\frac{1-f(s_{a_{j}})}{1+f(s_{a_{j}})}\right)}^{w_{j}}+1}\right\}$

$\underset{s_{a_{j}^{⁎}}\in m_{S}^{j^{⁎}}}{\cup }\left\{\frac{2\prod_{j=1}^{n}{\left(1+f(s_{a_{j}^{⁎}})\right)}^{w_{j}}-\prod_{j=1}^{n}{\left(1-f(s_{a_{j}^{⁎}})\right)}^{w_{j}}-\prod_{j=1}^{n}{\left(1+f(s_{a_{j}^{⁎}})\right)}^{wj}}{\prod_{j=1}^{n}{\left(1-f(s_{a_{j}^{⁎}})\right)}^{w_{j}}+\prod_{j=1}^{n}{\left(1+f(s_{a_{j}^{⁎}})\right)}^{w_{j}}}\right\}\geq$

$\underset{s_{a_{j}}\in m_{S}^{j}}{\cup }\left\{\frac{2\prod_{j=1}^{n}{\left(1+f(s_{a_{j}})\right)}^{w_{j}}-\prod_{j=1}^{n}{\left(1-f(s_{a_{j}^{⁎}})\right)}^{w_{j}}-\prod_{j=1}^{n}{\left(1+f(s_{a_{j}})\right)}^{wj}}{\prod_{j=1}^{n}{\left(1-f(s_{a_{j}^{⁎}})\right)}^{w_{j}}+\prod_{j=1}^{n}{\left(1+f(s_{a_{j}})\right)}^{w_{j}}}\right\}$

(3.3) $$f\;{}^{-1}\;\left(\underset{s_{a_{j}^{⁎}}\in m_{S}^{j^{⁎}}}{\cup }\left\{\frac{\prod_{j=1}^{n}{\left(1+f(s_{a_{j}})\right)}^{w_{j}}-\prod_{j=1}^{n}{\left(1-f(s_{a_{j}^{⁎}})\right)}^{w_{j}}}{\prod_{j=1}^{n}{\left(1-f(s_{a_{j}^{⁎}})\right)}^{w_{j}}+\prod_{j=1}^{n}{\left(1+f(s_{a_{j}^{⁎}})\right)}^{w_{j}}}\right\}\right)\geq f\;{}^{-1}\;\left(\underset{s_{a_{j}}\in m_{S}^{j}}{\cup }\left\{\frac{\prod_{j=1}^{n}{\left(1+f(s_{a_{j}})\right)}^{w_{j}}-\prod_{j=1}^{n}{\left(1-f(s_{a_{j}})\right)}^{w_{j}}}{\prod_{j=1}^{n}{\left(1-f(s_{a_{j}})\right)}^{w_{j}}+\prod_{j=1}^{n}{\left(1+f(s_{a_{j}})\right)}^{w_{j}}}\right\}\right)$$

Let $h(x_{1})=\frac{2-x_{1}}{x_{1}}$, $x_{1}\in (0,1]$, then $h^{\prime }(x_{1})=\frac{-2}{x_{1}^{2}}<0$, so, $h(x_{1})$ is decreasing function on (0,1]. If $f(s_{b_{j}})\geq f(s_{b_{j}^{⁎}})$, for all *j*, then $h(f(s_{b_{j}}))\leq h(f(s_{b_{j}^{⁎}})),j=1,2,...,n$. Therefore

$\underset{s_{b_{j}}\in n_{S}^{j}}{\cup }\left\{\frac{2-f(s_{b_{j}})}{f(s_{b_{j}})}\right\}\leq \underset{s_{b_{j}^{⁎}}\in n_{S}^{j^{⁎}}}{\cup }\left\{\frac{2-f(s_{b_{j}^{⁎}})}{f(s_{b_{j}^{⁎}})}\right\}$

$\Rightarrow \underset{s_{b_{j}}\in n_{S}^{j}}{\cup }\left\{\prod_{j=1}^{n}{\left(\frac{2-f(s_{b_{j}})}{f(s_{b_{j}})}\right)}^{w_{j}}\right\}\leq \underset{s_{b_{j}^{⁎}}\in n_{S}^{j^{⁎}}}{\cup }\left\{\prod_{j=1}^{n}{\left(\frac{2-f(s_{b_{j}^{⁎}})}{f(s_{b_{j}^{⁎}})}\right)}^{w_{j}}\right\}$

$\underset{s_{b_{j}^{⁎}}\in n_{S}^{j^{⁎}}}{\cup }\left\{\frac{1}{\prod_{j=1}^{n}{\left(\frac{2-f(s_{b_{j}^{⁎}})}{f(s_{b_{j}^{⁎}})}\right)}^{w_{j}}+1}\right\}\leq \underset{s_{b_{j}}\in n_{S}^{j}}{\cup }\left\{\frac{1}{\prod_{j=1}^{n}{\left(\frac{2-f(s_{b_{j}})}{f(s_{b_{j}})}\right)}^{w_{j}}+1}\right\}$

$\underset{s_{b_{j}^{⁎}}\in n_{S}^{j^{⁎}}}{\cup }\left\{\frac{2\prod_{j=1}^{n}{\left(f(s_{b_{j}^{⁎}})\right)}^{w_{j}}}{\prod_{j=1}^{n}{\left(2-f(s_{b_{j}^{⁎}})\right)}^{wj}+\prod_{j=1}^{n}{\left(f(s_{b_{j}^{⁎}})\right)}^{w_{j}}}\right\}\leq \underset{s_{b_{j}}\in n_{S}^{j}}{\cup }\left\{\frac{2\prod_{j=1}^{n}{\left(f(s_{b_{j}})\right)}^{w_{j}}}{\prod_{j=1}^{n}{\left(2-f(s_{b_{j}})\right)}^{w_{j}}+\prod_{j=1}^{n}{\left(f(s_{b_{j}})\right)}^{w_{j}}}\right\}$

(3.4) $$f^{-1}\left(\underset{s_{b_{j}^{⁎}}\in n_{S}^{j^{⁎}}}{\cup }\left\{\frac{2\prod_{j=1}^{n}{\left(f(s_{b_{j}^{⁎}})\right)}^{w_{j}}}{\prod_{j=1}^{n}{\left(2-f(s_{b_{j}^{⁎}})\right)}^{w_{j}}+\prod_{j=1}^{n}{\left(f(s_{b_{j}^{⁎}})\right)}^{w_{j}}}\right\}\right)\leq f^{-1}\left(\underset{s_{b_{j}}\in n_{S}^{j}}{\cup }\left\{\frac{2\prod_{j=1}^{n}{\left(f(s_{b_{j}})\right)}^{w_{j}}}{\prod_{j=1}^{n}{\left(2-f(s_{b_{j}})\right)}^{w_{j}}+\prod_{j=1}^{n}{\left(f(s_{b_{j}})\right)}^{w_{j}}}\right\}\right)$$

Let HIFLEWA(IS⁎,...,IS⁎)=(f(∪sμj⁎∈mSj⁎{f(sμj⁎)})−1,f−1(∪sνj⁎∈nSj⁎{f(sνj⁎)})) and HIFLEWA(IS1,...,ISn)=(f(∪sμj∈mSj{  f(sμj)})−1,f−1(∪sνj∈nSj{f(sνj)})), then from the inequalities (3.3) and (3.4) and the inequalities $HIFLEWA(I_{S}^{1},...,I_{S}^{n})$ $\leq HIFLEWA(I_{S}^{⁎},...,I_{S}^{⁎})$ hold. □

Definition 3.5 Let $\left(I_{S}^{1},I_{S}^{2},...I_{S}^{n}\right)$ be a set of HIFLEs and $w={\left(w_{1},w_{2},...w_{n}\right)}^{t}$ be a weight vector of ${\left\{I_{S}^{j}\right\}}_{j=1}^{n}$ with $0\leq w_{j}\leq 1$ such that $\underset{j=1}{\overset{n}{\sum w_{j}}}=1$. Then the hesitant intuitionistic fuzzy linguistic Einstein weighted averaging (HIFLEWA) operator is defined as follows:

$$(I_{S}^{1},I_{S}^{2},...I_{S}^{n})=\underset{j=1}{\overset{n}{⊕}}(w_{j}I_{S}^{j}).$$

Theorem 3.6 *Let* $I_{j}=\left\{m_{S_{j}},n_{S_{j}}\right\}$ *where* $m_{S_{j}}=\left\{s_{a_{j}}{|}_{a_{j}}\epsilon [0,g]\right\}$ *and* $n_{S_{j}}=\left\{s_{b_{j}}{|}_{b_{j}}\epsilon [0,g]\right\},(j=1,2,3,...n)$ *be a collection of HIFLEs, then*

HIFLEWA(IS1,IS2,...ISn)=(f(∪saj∈mSj{∏j=1n(1+f(saj))  wj  −  ∏j=1n(1−f(saj))wj  ∏j=1n(1+f(saj))wj  +  ∏j=1n(1−f(saj))wj})−1,

$f^{-1}\left(\underset{s_{b_{j}}\in n_{S_{j}}}{\cup }\left\{\frac{2\prod_{j=1}^{n}\;{\left(f(s_{b_{j}})\right)}^{w_{j}}}{\prod_{j=1}^{n}{\left(2-f(s_{b_{j}})\right)}^{w_{j}}+\;\prod_{j=1}^{n}{\left(\;f(s_{b_{j}})\right)}^{w_{j}}}\right\}\right))$

Proof By Utilizing the operational laws defined for HIFLTSs $I_{S}^{1}=\left\{m_{S}^{1},n_{S}^{1}\right\}$ and $I_{S}^{2}=\left\{m_{S}^{2},n_{S}^{2}\right\}$ as discussed in Definition in 3.1, we have

w1I1=(f(∪sa1∈mS1{(1+f(sa1))  w1  −  (1−f(sa1))w1  (1+f(sa1))w1  +  (1−f(sa1))w1})−1,f−1(∪sb1∈nS1{2  (f(sb1))w1(2−f(sb1))w1+  (f(sb1))w1}))

w2I2=(f(∪sa2∈mS2{(1+f(sa2))  w2  −  (1−f(sa2))w2  (1+f(sa2))w2  +  (1−f(sa2))w2})−1,f−1(∪sb2∈nS2{2  (f(sb2))w2(2−f(sb2))w2+  (f(sb2))w2}))

By using mathematical induction, we have for n=2,

$w_{1}I_{1}+w_{2}I_{2}=$

f(∪sa1∈mS1{(1+f(sa1))  w1  −  (1−f(sa1))w1  (1+f(sa1))w1  +  (1−f(sa1))w1})−1⊕(f(∪sa2∈mS2{(1+f(sa2))  w2  −  (1−f(sa2))w2  (1+f(sa2))w2  +  (1−f(sa2))w2})−1,

$f^{-1}\left(\underset{s_{b_{1}}\in n_{S}^{1}}{\cup }\left\{\frac{2\;{\left(f(s_{b_{1}})\right)}^{w_{1}}}{{\left(2-f(s_{b_{1}})\right)}^{w_{1}}+\;{\left(\;f(s_{b_{1}})\right)}^{w1}}\right\}\right))⊗f^{-1}\left(\underset{s_{b_{2}}\in n_{S}^{2}}{\cup }\left\{\frac{2\;{\left(f(s_{b_{2}})\right)}^{w_{2}}}{{\left(2-f(s_{b_{2}})\right)}^{w_{2}}+\;{\left(\;f(s_{b_{2}})\right)}^{w2}}\right\}\right)$

=(f(∪saj∈mSj{∏j=1n(1+f(saj))  wj  −  ∏j=1n(1−f(saj))wj  ∏j=1n(1+f(saj))wj  +  ∏j=1n(1−f(saj))wj})−1,

$f^{-1}\left(\underset{s_{b_{j}}\in n_{S_{j}}}{\cup }\left\{\frac{2\prod_{j=1}^{n}\;{\left(f(s_{b_{j}})\right)}^{w_{j}}}{\prod_{j=1}^{n}{\left(2-f(s_{b_{j}})\right)}^{w_{j}}+\;\prod_{j=1}^{n}{\left(\;f(s_{b_{j}})\right)}^{w_{j}}}\right\}\right))$

The conditions $\max(I_{S}^{1})+\min(I_{S}^{2})\leq g$ and $\min(I_{S}^{1})+\max(I_{S}^{2})\leq g$ can be easily proved and always hold.

Let the theorem hold for n=k, i.e.

=(f(∪saj∈mSj{∏j=1k(1+f(saj))  wj  −  ∏j=1k(1−f(saj))wj  ∏j=1k(1+f(saj))wj  +  ∏j=1k(1−f(saj))wj})−1,

$f^{-1}\left(\underset{s_{b_{j}}\in n_{S_{j}}}{\cup }\left\{\frac{2\prod_{j=1}^{k}\;{\left(f(s_{b_{j}})\right)}^{w_{j}}}{\prod_{j=1}^{k}{\left(2-f(s_{b_{j}})\right)}^{w_{j}}+\;\prod_{j=1}^{k}{\left(\;f(s_{b_{j}})\right)}^{w_{j}}}\right\}\right))$

For proving it for n=k+1, suppose

=(f(∪saj∈mSj{∏j=1k(1+f(saj))  wj  −  ∏j=1k(1−f(saj))wj  ∏j=1k(1+f(saj))wj  +  ∏j=1k(1−f(saj))wj})−1,

$f^{-1}\left(\underset{s_{b_{j}}\in n_{S_{j}}}{\cup }\left\{\frac{2\prod_{j=1}^{k}\;{\left(f(s_{b_{j}})\right)}^{w_{j}}}{\prod_{j=1}^{k}{\left(2-f(s_{b_{j}})\right)}^{w_{j}}+\;\prod_{j=1}^{k}{\left(\;f(s_{b_{j}})\right)}^{w_{j}}}\right\}\right))⊕w_{1}I_{1}$

=(f(∪saj∈mSj{∏j=1k(1+f(saj))  wj  −  ∏j=1k(1−f(saj))wj  ∏j=1k(1+f(saj))wj  +  ∏j=1k(1−f(saj))wj})−1,

$f^{-1}\left(\underset{s_{b_{j}}\in n_{S_{j}}}{\cup }\left\{\frac{2\prod_{j=1}^{k}\;{\left(f(s_{b_{j}})\right)}^{w_{j}}}{\prod_{j=1}^{k}{\left(2-f(s_{b_{j}})\right)}^{w_{j}}+\;\prod_{j=1}^{k}{\left(\;f(s_{b_{j}})\right)}^{w_{j}}}\right\}\right))⊕$

(f(∪sa1∈mS1{(1+f(sa1))  w1  −  (1−f(sa1))w1  (1+f(sa1))w1  +  (1−f(sa1))w1})−1,f−1(∪sb1∈nS1{2  (f(sb1))w1(2−f(sb1))w1+  (f(sb1))w1}))

=(f(∪saj∈mSj{∏j=1k+1(1+f(saj))  wj  −  ∏j=1k+1(1−f(saj))wj  ∏j=1k+1(1+f(saj))wj  +  ∏j=1k+1(1−f(saj))wj})−1,

$f^{-1}\left(\underset{s_{b_{j}}\in n_{S_{j}}}{\cup }\left\{\frac{2\prod_{j=1}^{k+1}\;{\left(f(s_{b_{j}})\right)}^{w_{j}}}{\prod_{j=1}^{k+1}{\left(2-f(s_{b_{j}})\right)}^{w_{j}}+\;\prod_{j=1}^{k+1}{\left(\;f(s_{b_{j}})\right)}^{w_{j}}}\right\}\right))$

The theorem holds for n=k+1, hence by principle of mathematical induction, it also holds for all $n\in N^{+}$.

In particular, if $w={\left(\frac{1}{n},...,\frac{1}{n}\right)}^{T}$ such that $\sum_{j=1}^{n}w_{j}=1$. Then, the HIFLEWA operator reduces to hesitant intuitionistic linguistic Einstein averaging operator as follows:

HIFLEA(IS1,IS2,...ISn)=1n[f(∪saj∈mSj{∏j=1n(1+f(saj))  −  ∏j=1n(1−f(saj))  ∏j=1n(1+f(saj))  +  ∏j=1n(1−f(saj))})−1,

$f^{-1}\left(\underset{s_{b_{j}}\in n_{S_{j}}}{\cup }\left\{\frac{2\prod_{j=1}^{n}\;(f(s_{b_{j}}))}{\prod_{j=1}^{n}(2-f(s_{b_{j}}))+\;\prod_{j=1}^{n}(\;f(s_{b_{j}}))}\right\}\right)]$. □

## 4 The proposed HIFLTSs EDAS method

A MCGDM problem with HIFLTS is presented in this section based on EDAS approach. Suppose $A=\left\{A_{1},A_{2},...,A_{r}\right\}$ be the required set for all the alternatives and $D=\left\{D_{1},D_{2},...,D_{k}\right\}$ be the required set for all the DMs. The alternatives are evaluated by the DMs as for a few criteria $c_{j}(j=1,2,...,n)$. Let $w={\left(w_{1},w_{2},...,w_{n}\right)}^{T}$ be the weighting vector of the criteria, which holds $\overset{n}{\sum_{j=1}}w_{j}=1$, where $0\leq w_{j}\leq 1,\;j=1,2,...,n$. With the help of the foregoing assumptions, the EDAS method is carried out in nine steps:

**Step 1.** The hesitant intuitionistic fuzzy linguistic judgement matrices are generated using expert opinion.

DMs frequently express their opinions in the form of words or linguistic data. Converting such linguistic data into HIFLTSs can be done using context-free grammar [35]. Let $I_{s}^{t}(t=1,2,...,k)$ be a judgement matrix that contains HIFLTSs data as follows:

$$I_{s}^{t}=\left[\begin{array}{cccc} I_{s_{11}}^{t}\; & I_{s_{12}}^{t}\; & \cdots \; & I_{s_{1n}}^{t}\\ I_{s_{21}}^{t}\; & I_{s_{22}}^{t}\; & \cdots \; & I_{s_{2n}}^{t}\\ \vdots \; & \vdots \; & \ddots \; & \vdots \\ I_{s_{r1}}^{t}\; & I_{s_{r2}}^{t}\; & \cdots \; & I_{s_{rn}}^{t} \end{array}\right]$$

Each $I_{s_{ij}}^{t}=\left\{m_{s_{ij}}^{t},n_{s_{ij}}^{t}\right\}$ is an HIFLTS value reflecting the degree to which the alternative $A_{i}$ meets the criteria $c_{j}$. Here $m_{s_{ij}}^{t}=\cup_{s_{a_{l}^{ij}\in m_{s_{ij}}^{t}}\;}\left(s_{a_{l}^{ij}}|l=1,2,...,\neq m_{s_{ij}}^{t}\right)$ and $n_{s_{ij}}^{t}=\cup_{s_{b_{p}^{ij}\in m_{s_{ij}}^{t}}\;}\left(s_{b_{p}^{ij}}|p=1,2,...,\neq n_{s_{ij}}^{t}\right)$.

**Step 2.** Using the aggregation rules suggested in Definition 3.5, the construction of aggregated hesitant intuitionistic fuzzy linguistic judgement matrix $I_{s}$ is carried out in this stage. The following is the definition of the designed $I_{s}$ matrix:

$$I_{s}=\left[\begin{array}{cccc} I_{s_{11}}\; & I_{s_{12}}\; & \cdots \; & I_{s_{1n}}\\ I_{s_{21}}\; & I_{s_{22}}\; & \cdots \; & I_{s_{2n}}\\ \vdots \; & \vdots \; & \ddots \; & \vdots \\ I_{s_{r1}}\; & I_{s_{r2}}\; & \cdots \; & I_{s_{rn}} \end{array}\right],$$

where each $I_{s_{ij}}=\left\{m_{s_{ij}},n_{s_{ij}}\right\}$ is HIFLTS, $m_{s_{ij}}=\cup_{s_{a_{l}^{ij}\in m_{s_{ij}}^{t}}}\left(s_{a_{l}^{ij}}|l=1,2,...,\neq m_{s_{ij}}\right)$ and $n_{s_{ij}}=\cup_{s_{b_{p}^{ij}\in m_{s_{ij}}^{t}}}\left(s_{b_{p}^{ij}}|p=1,2,...,\neq n_{s_{ij}}\right)$.

**Step 3.** Determine the HIFLTS average decision matrix using the criteria mentioned below.

$AV_{i}=HIFLEA(I_{S}^{1},I_{S}^{2},...I_{S}^{n})=\frac{1}{n}\overset{k}{\underset{j=1}{⊕}}I_{S}^{j}=$

1n[(f(∪saj∈mSj{∏j=1k(1+f(saj))  −  ∏j=1k(1−f(saj))  ∏j=1k(1+f(saj))  +  ∏j=1k(1−f(saj))})−1,f−1(∪sbj∈nSj{2∏j=1n  (f(sbj))∏j=1n(2−f(sbj))+  ∏j=1n(f(sbj))}))]

**Step 4.** Determine the score (Sc) function for the aggregated matrix. Let $I_{S}=\left\{m_{S},n_{S}\right\}$ be a HIFLE, then the score function for the aggregated matrix defined as follows $Sc(I_{S})=\frac{1}{g}$ $\sum_{m_{S}^{i}\epsilon ms_{ij}}\frac{m_{S}^{i}}{\#m_{S}^{i}}-\frac{1}{g}$ $\sum_{n_{S}^{i}\epsilon ns_{ij}}\frac{n_{S}^{i}}{\#n_{S}^{i}}$, where $\#m_{S}^{i}$ and $\#n_{S}^{i}$ are the number of elements in $m_{S}^{i}$ and $n_{S}^{i}$ respectively. Based on step 3, we also determine the score of average matrix $Sc(AV_{i})$, which is a matrix ${\left[AV_{i}\right]}_{5\times 1}$.

**Step 5.** Determine the positive distance from average (PDA) and the negative distance from average (NDA) for all the alternatives, as follows:

$$PDA={\left[PDA_{ij}\right]}_{5\times 4},NDA={\left[NDA_{ij}\right]}_{5\times 4}\;PDA_{ij}=\frac{\max\left(0,Sc(I_{S})-Sc(AV_{i})\right)}{Sc(AV_{i})},\;NDA_{ij}=\frac{\max\left(0,Sc(AV_{i})-Sc(I_{S})\right)}{Sc(AV_{i})}$$

When we determine the $PDA_{ij}$ or $NDA_{ij}$, when $\frac{Sc(I_{S})-Sc(AV_{i})}{Sc(AV_{i})}$ or $\frac{Sc(AV_{i})-Sc(I_{S})}{Sc(AV_{i})}$ give us negative value then we replace it with 0.

**Step 6.** Determine the weighted summation (SP) for $\left(PDA_{ij}\right)$ and (SN) for $\left(NDA_{ij}\right)$ for all the alternatives, as follows:

$$SP_{i}=\overset{n}{\sum_{j=1}}w_{j}⁎PDA_{ij},\;SN_{i}=\overset{n}{\sum_{j=1}}w_{j}⁎NDA_{ij}$$

**Step 7.** Determine the normalized values (NSP) and (NSN) by utilizing $SP_{i}$ and $SN_{i}$ for all alternatives, as follows:

$$NSP_{i}=\frac{SP_{i}}{Max_{i}\left(SP_{i}\right)}\text{, }NSN_{i}=1-\frac{SN_{i}}{Max_{i}\left(SN_{i}\right)}$$

**Step 8.** Determine the appraisal score $ASc_{i}\left(0\leq ASc_{i}\leq 1\right)$ for all alternatives, as follows:

$$ASc_{i}=\frac{NSP_{i}+NSNA_{i}}{2}$$

The alternatives are arranged in decreasing values of $ASc_{i}$ for all $A_{i}$. The $A_{i}$ with largest value will be declared the best one from all the alternatives.

## 5 A numerical example

In this Section, a numerical model based on HIFLTSs is being used to demonstrate the procedure's adequacy and practical benefits.

Due to recent government initiatives surrounding the Financial Action Task Force rules, a multinational business wants to make a large investment in Pakistan in the current situation. The trader company wants to guarantee their future by purchasing the best insurance coverage. The company of traders has received four suggestions as alternatives

• $A_{1}$: National Insurance Corporation,
• $A_{2}$: Pakistan Reinsurance Company Limited,
• $A_{3}$: State Life Insurance Corporation Limited and
• $A_{4}$: Postal Life Insurance.

The four insurance companies have made their decisions based on five criteria:

• $c_{1}-$ social burden,
• $c_{2}-$ cash value,
• $c_{3}-$ tax benefits,
• $c_{4}-$ lack of trust, and
• $c_{5}-$ flexibility.

Three DMs $D_{1}$, $D_{2}$ and $D_{3}$ are mentioned to assess the four proposals by using a set of linguistic terms as follows:

Let $S=\left\{s_{0},s_{1},s_{2},s_{3},s_{4},s_{5},s_{6}\right\}=\{\text{extremely poor, very poor, poor, medium, good, very good, extremely good}\}$ be the required linguistic term set. Let us consider the weight vector of the criteria is to be w=(0.10,0.50,0.20,0.10,0.10).

**Step 1**. The three experts express their thoughts in linguistic form regarding the performance of $A_{i}\;(i=1,2,3,4)$ on the criteria $c_{j}\;(j=1,2,3,4,5)$ using the intuitionistic fuzzy linguistic decision matrices illustrated in Table 1, Table 2, Table 3.

**Table 1:** Decision matrix $I_{s}^{1}$ provided by *DM*_1_.

	*A*_1_	*A*_2_	*A*_3_	*A*_4_
*c*_1_	{(*s*_0_,*s*_1_),(*s*_3_,*s*_4_)}	$\left\{\left(s_{3},s_{4}\right),\left(s_{2}\right)\right\}$	$\left\{\left(s_{4},s_{5}\right),\left(s_{1}\right)\right\}$	$\left\{\left(s_{6}\right),\left(s_{0}\right)\right\}$
*c*_2_	$\left\{\left(s_{3},s_{4}\right),\left(s_{1},s_{2}\right)\right\}$	$\left\{\left(s_{0},s_{1},s_{2}\right),\left(s_{2},s_{3}\right)\right\}$	$\left\{\left(s_{4},s_{5}\right),\left(s_{0},s_{1}\right)\right\}$	$\left\{\left(s_{3},s_{4}\right),\left(s_{2}\right)\right\}$
*c*_3_	$\left\{\left(s_{5}\right),\left(s_{0},s_{1}\right)\right\}$	$\left\{\left(s_{4},s_{5}\right),\left(s_{1}\right)\right\}$	$\left\{\left(s_{3},s_{4}\right),\left(s_{0},s_{1}\right)\right\}$	$\left\{\left(s_{0},s_{1},s_{2}\right),\left(s_{3},s_{4}\right)\right\}$
*c*_4_	$\left\{\left(s_{2}\right),\left(s_{2},s_{3}\right)\right\}$	$\left\{\left(s_{2},s_{3}\right),\left(s_{1},s_{2}\right)\right\}$	$\left\{\left(s_{3},s_{4}\right),\left(s_{2}\right)\right\}$	$\left\{\left(s_{3}\right),\left(s_{2},s_{3}\right)\right\}$
*c*_5_	$\left\{\left(s_{1},s_{2}\right),\left(s_{2},s_{3}\right)\right\}$	$\left\{\left(s_{0},s_{1}\right),\left(s_{3},s_{4},s_{5}\right)\right\}$	$\left\{\left(s_{4}\right),\left(s_{2}\right)\right\}$	$\left\{\left(s_{4},s_{5}\right),\left(s_{0},s_{1}\right)\right\}$

**Table 2:** Decision matrix $I_{s}^{2}$ provided by *DM*_2_.

	*A*_1_	*A*_2_	*A*_3_	*A*_4_
*c*_1_	{(*s*_3_,*s*_4_),(*s*_1_,*s*_2_)}	$\left\{\left(s_{6}\right),\left(s_{0}\right)\right\}$	$\left\{\left(s_{0},s_{1}\right),\left(s_{3},s_{4}\right)\right\}$	$\left\{\left(s_{4}\right),\left(s_{2}\right)\right\}$
*c*_2_	$\left\{\left(s_{2},s_{3},s_{4}\right),\left(s_{1},s_{2}\right)\right\}$	$\left\{\left(s_{2},s_{3}\right),\left(s_{2}\right)\right\}$	$\left\{\left(s_{0},s_{1}\right),\left(s_{4},s_{5}\right)\right\}$	$\left\{\left(s_{2},s_{3},s_{4}\right),\left(s_{1},s_{2}\right)\right\}$
*c*_3_	$\left\{\left(s_{1},s_{2}\right),\left(s_{2},s_{3}\right)\right\}$	$\left\{\left(s_{0},s_{1}\right),\left(s_{4}\right)\right\}$	$\left\{\left(s_{2},s_{3}\right),\left(s_{1},s_{2,}s_{3}\right)\right\}$	$\left\{\left(s_{1},s_{2}\right),\left(s_{0},s_{1}\right)\right\}$
*c*_4_	$\left\{\left(s_{5}\right),\left(s_{1}\right)\right\}$	$\left\{\left(s_{3},s_{4}\right),(s_{1},s_{2})\right\}$	$\left\{\left(s_{4},s_{5}\right),\left(s_{1}\right)\right\}$	$\left\{\left(s_{0},s_{1}\right),\left(s_{5}\right)\right\}$
*c*_5_	$\left\{\left(s_{2},s_{3}\right),\left(s_{1},s_{2,}s_{3}\right)\right\}$	$\left\{\left(s_{1},s_{2}\right),\left(s_{2},s_{3}\right)\right\}$	$\left\{\left(s_{4},s_{5}\right),\left(s_{0},s_{1}\right)\right\}$	$\left\{\left(s_{3},s_{4}\right),\left(s_{1},s_{2}\right)\right\}$

**Table 3:** Decision matrix $I_{s}^{3}$ provided by *DM*_3_.

	*A*_1_	*A*_2_	*A*_3_	*A*_4_
*c*_1_	{(*s*_1_,*s*_2_,*s*_3_),(*s*_2_,*s*_3_)}	$\left\{\left(s_{3},s_{4}\right),\left(s_{2}\right)\right\}$	$\left\{\left(s_{0},s_{1}\right),\left(s_{3},s_{4}\right)\right\}$	$\left\{\left(s_{3},s_{4},s_{5}\right),\left(s_{1}\right)\right\}$
*c*_2_	$\left\{\left(s_{1}\right),\left(s_{4},s_{5}\right)\right\}$	$\left\{\left(s_{3},s_{4}\right),\left(s_{2}\right)\right\}$	$\left\{\left(s_{5}\right),\left(s_{1}\right)\right\}$	$\left\{\left(s_{4},s_{5}\right),\left(s_{0},s_{1}\right)\right\}$
*c*_3_	$\left\{\left(s_{2},s_{3}\right),\left(s_{1},s_{2}\right)\right\}$	$\left\{\left(s_{1},s_{2},s_{3}\right),\left(s_{2},s_{3}\right)\right\}$	$\left\{\left(s_{4},s_{5}\right),\left(s_{0},s_{1}\right)\right\}$	$\left\{\left(s_{1},s_{2}\right),\left(s_{2},s_{3}\right)\right\}$
*c*_4_	$\left\{\left(s_{6}\right),\left(s_{0}\right)\right\}$	$\left\{\left(s_{2},s_{3},s_{4}\right),\left(s_{1},s_{2}\right)\right\}$	$\left\{\left(s_{0},s_{1},s_{2}\right),\left(s_{4}\right)\right\}$	$\left\{\left(s_{4},s_{5}\right),\left(s_{0},s_{1}\right)\right\}$
*c*_5_	$\left\{\left(s_{3},s_{4}\right),\left(s_{1},s_{2}\right)\right\}$	$\left\{\left(s_{3}\right),\left(s_{2},s_{3}\right)\right\}$	$\left\{\left(s_{2},s_{3}\right),\left(s_{1},s_{2}\right)\right\}$	$\left\{\left(s_{4},s_{5}\right),\left(s_{0},s_{1}\right)\right\}$

**Step 2**. $H={\left[H_{ij}\right]}_{5\times 4}$ is the aggregated hesitant intuitionistic fuzzy linguistic judgement matrix, where,

$$H=\left[\begin{array}{cccc} H_{1}^{1}\; & H_{1}^{2}\; & H_{1}^{3}\; & H_{1}^{4}\\ H_{2}^{1}\; & H_{2}^{2}\; & H_{2}^{3}\; & H_{2}^{4}\\ H_{3}^{1}\; & H_{3}^{2}\; & H_{3}^{3}\; & H_{3}^{4}\\ H_{4}^{1}\; & H_{4}^{2}\; & H_{4}^{3}\; & H_{4}^{4}\\ H_{5}^{1}\; & H_{5}^{2}\; & H_{5}^{3}\; & H_{5}^{4} \end{array}\right]$$

$$H_{1}^{1}=\left\{\left(s_{4.8191}\right),\left(s_{0.0723},s_{0.1081},s_{0.1200},s_{0.1579},s_{0.1791,}s_{0.2353},s_{0.2609},s_{0.3871}\right)\right\}$$

$$H_{2}^{1}=\left\{\begin{array}{c} \left(s_{4.7234},s_{5.1176},s_{5.2000},s_{5.4545},s_{5.6667}\right),(s_{0.0494},s_{0.0704},s_{0.1081},s_{0.1538},\\ s_{0.2353},s_{0.3333}) \end{array}\right\}$$

$$H_{3}^{1}=\left\{\left(s_{5.6226,}s_{5.7333},s_{5.7458},s_{5.8209}\right),\left(s_{0},s_{0.0198},s_{0.0330},s_{0.0435},s_{0.0723}\right)\right\}$$

$$H_{4}^{1}=\left\{\left(s_{6}\right),\left(s_{0}\right)\right\}$$

$$H_{5}^{1}=\left\{\begin{array}{c} \left(s_{4.7234},s_{5.0769},s_{5.1176},s_{5.2000},s_{5.3684},s_{5.4286},s_{5.4545},s_{5.6129}\right),\\ \left(s_{0.0198},s_{0.0330},s_{0.0435},s_{0.0723},s_{0.0723},s_{0.0952},s_{0.1200},s_{0.1579},s_{0.2609}\right) \end{array}\right\}$$

$$H_{1}^{2}=\left\{\left(s_{6}\right),\left(s_{0}\right)\right\}$$

$$H_{2}^{2}=\left\{\begin{array}{c} (s_{4.2857},s_{4.7234},s_{4.8000},s_{4.9091},s_{5.0769},s_{5.1176},s_{5.2000},s_{5.2500},s_{5.3684},\\ s_{5.4286},s_{5.4545},s_{5.6129}),\left(s_{0.0952},s_{0.1579}\right) \end{array}\right\}$$

$$H_{3}^{2}=\left\{\begin{array}{c} (s_{4.5000},s_{4.8889},s_{4.9091},s_{5.2000},s_{5.2500},s_{5.2683},s_{5.4545},s_{5.4681},s_{5.4783},\\ s_{5.6226},s_{5.6471},s_{5.7458}),\left(s_{0.1081},s_{0.1791}\right) \end{array}\right\}$$

$$H_{4}^{2}=\left\{\begin{array}{c} (s_{5.0769},s_{5.3684},s_{5.3684},s_{5.4286},s_{5.5714},s_{5.6129},s_{5.6129},s_{5.7391},s_{5.7647},\\ s_{5.8421}),\left(s_{0.0090},s_{0.0198},s_{0.0435},s_{0.0952}\right) \end{array}\right\}$$

$$H_{5}^{2}=\left\{\begin{array}{c} \left(s_{3.6923},s_{4.2558},s_{4.2857},s_{4.7234}\right),(s_{0.1579},s_{0.2353},s_{0.2609},s_{0.3333},\\ s_{0.3871},s_{0.4286},s_{0.5455},s_{0.6316},s_{0.8824}) \end{array}\right\}$$

$$H_{1}^{3}=\left\{\left(s_{4.0000},s_{4.5000},s_{4.8889},s_{5.0000},s_{5.2683},s_{5.4681}\right),\left(s_{0.1200},s_{0.1791},s_{0.2667}\right)\right\}$$

$$H_{2}^{3}=\left\{\left(s_{5.7857},s_{5.8462},s_{5.9016},s_{5.9296}\right),\left(s_{0},s_{0.0494},s_{0.0704}\right)\right\}$$

$$H_{3}^{3}=\left\{\begin{array}{c} \left(s_{5.6129},s_{5.7391},s_{5.7647},s_{5.8209},s_{5.8421},s_{5.8800},s_{5.8919},s_{5.9277}\right),(s_{0},\\ s_{0.0090},s_{0.0198},s_{0.0330}) \end{array}\right\}$$

$$H_{4}^{3}=\left\{\begin{array}{c} (s_{5.2500},s_{5.4545},s_{5.5385},s_{5.6129},s_{5.6471},s_{5.6667},s_{5.7458},s_{5.7647},s_{5.7857},\\ s_{5.8209},s_{5.8462},s_{5.8919}),\left(s_{0.1081}\right) \end{array}\right\}$$

$$H_{5}^{3}=\left\{\left(s_{5.7647},s_{5.8421},s_{5.8919},s_{5.9277}\right),\left(s_{0},s_{0.0198},s_{0.0435}\right)\right\}$$

$$H_{1}^{4}=\left\{\left(s_{6}\right),\left(s_{0}\right)\right\}$$

$$H_{2}^{4}=\left\{\begin{array}{c} (s_{5.6129},s_{5.7391},s_{5.7647},s_{5.8209},s_{5.8421},s_{5.8800},s_{5.8919},s_{5.9048},s_{5.9277},\\ s_{5.9565}),\left(s_{0},s_{0.0198},s_{0.0435}\right) \end{array}\right\}$$

$$H_{3}^{4}=\left\{\begin{array}{c} \left(s_{1.9459},s_{2.7949},s_{2.8421},s_{3.5610},s_{3.6000},s_{4.1818},s_{4.6667}\right),(s_{0},s_{0.0723},\\ s_{0.1081},s_{0.1200},s_{0.1791}) \end{array}\right\}$$

$$H_{4}^{4}=\left\{\left(s_{5.2500,}s_{5.4545},s_{5.6471},s_{5.7458}\right),\left(s_{0},s_{0.1538},s_{0.2542}\right)\right\}$$

$$H_{5}^{4}=\left\{\left(s_{5.8421,}s_{5.9048},s_{5.9277},s_{5.9565},s_{5.9670},s_{5.9802}\right),\left(s_{0},s_{0.0090},s_{0.0198}\right)\right\}$$

**Step 3.** The average decision matrix for HIFLTS is as follows:

$$AV_{i}=\left[\begin{array}{c} \left\{\left(s_{6}\right),\left(s_{0}\right)\right\}\\ \left\{\left(s_{3.8},s_{3.9},s_{4},s_{4.1},s_{4.2},s_{4.3},s_{4.4},s_{4.5},s_{4.6},s_{4.7},s_{4.8},s_{4.9},s_{4.9},s_{5},s_{5.1},s_{5.2},s_{5.3}\right),\left(s_{0}\right)\right\}\\ \left\{\left(s_{4},s_{4.1},s_{4.2},s_{4.3},s_{4.4},s_{4.5},s_{4.6},s_{4.7},s_{4.8},s_{4.9},s_{5},s_{5.1},\right),\left(s_{0},s_{0.2},s_{0.3},s_{0.4}\right)\right\}\\ \left\{\left(s_{4.7},s_{4.8},s_{4.9},s_{5},s_{5.1},s_{5.2},s_{5.3}\right),\left(s_{0},s_{0.2},s_{0.3},s_{0.4}\right)\right\}\\ \left\{\left(s_{4.3},s_{4.4},s_{4.5},s_{4.6},s_{4.7},s_{4.8},s_{4.9},s_{5},s_{5.1},s_{5.2},s_{5.3}\right),\left(s_{0},s_{0.2},s_{0.3},s_{0.4},s_{0.5},s_{0.6}\right)\right\} \end{array}\right]$$

**Step 4.**Table 4 shows the score values for the aggregated matrix and the average decision matrix.

**Table 4:** Score values of the aggregated matrix *H* and *AV*_*i*_ matrix.

	*A*_1_	*A*_2_	*A*_3_	*A*_4_	*Sc*(*AV*_*i*_)
*c*_1_	0.7715	1.0000	0.7776	1.0000	1.0000
*c*_2_	0.8457	0.8293	0.9710	0.9688	0.7749
*c*_3_	0.9495	0.8571	0.9668	0.5457	0.7159
*c*_4_	1.0000	0.9192	0.9268	0.8981	0.7939
*c*_5_	0.8584	0.6358	0.9726	0.9873	0.7464

**Step 5.**Table 5, Table 6 provide the PDA and NDA values for all of the alternatives.

**Table 5:** The *PDA*_*ij*_ values for all *A*_*i*_.

	*A*_1_	*A*_2_	*A*_3_	*A*_4_
*c*_1_	0	0	0	0
*c*_2_	0.0913	0.0701	0.2530	0.2502
*c*_3_	0.3264	0.1973	0.3491	0
*c*_4_	0.2596	0.1578	0.1674	0.1312
*c*_5_	0.1500	0	0.3030	0.3227

**Table 6:** The *NDA*_*ij*_ values for all *A*_*i*_.

	*A*_1_	*A*_2_	*A*_3_	*A*_4_
*c*_1_	0.2285	0	0.2224	0
*c*_2_	0	0	0	0
*c*_3_	0	0	0	0.2376
*c*_4_	0	0	0	0
*c*_5_	0	0.1482	0	0

**Step (7-8)**. Table 7 lists the $SP_{i}$, $SN_{i}$, $NSP_{i}$, $NSN_{i}$ and $ASc_{i}$ values as well as their ranking for each alternative.

**Table 7:** The *SP*_*i*_, *SN*_*i*_, *NSP*_*i*_, *NSN*_*i*_, *ASc*_*i*_ and ranking.

	*SP*_*i*_	*SN*_*i*_	*NSP*_*i*_	*NSN*_*i*_	*ASc*_*i*_	Ranking
*A*_1_	0.1519	0.0228	0.6242	0.5192	0.5717	2
*A*_2_	0.0903	0.0148	0.3711	0.8882	0.5296	3
*A*_3_	0.2433	0.0222	1.0000	0.5321	0.7660	1
*A*_4_	0.1705	0.0475	0.7006	0	0.3503	4

The ranking $\left(A_{3}≻A_{1}≻A_{2}≻A_{4}\right)$ of the alternatives obtained from EDAS shows the aftereffects of the coordinated technique. As a result, the most appropriate outcome for an insurance policy is identified as $A_{3}$ as the best option. As a result, the trader company is encouraged to choose $A_{3}$ as the best alternative and guarantee their future through an insurance policy.

### 5.1 Comparison Analysis

By considering the same numerical problem, the effectiveness of alternatives achieved with the EDAS approach can be checked with the existing TOPSIS method. The (HIFLEWA) operator will be used to aggregate decision matrices, as it is in the EDAS technique.

As in [14], the hesitant intuitionistic linguistic positive ideal solution $H_{j^{+}}$ and negative ideal solution $H_{j^{-}}$ are produced. $H_{j^{+}}$ and $H_{j^{-}}$ are HIFLTS that only have a single linguistic term in the membership and non-membership functions, respectively. In above example $c_{2},\;c_{3}$ and $c_{5}$ are benefits criteria while $c_{1}$ and $c_{4}$ are cost criteria.

$$H_{j}^{+}=[\left\{\left(s_{4}\right),\left(s_{0.3871}\right)\right\},\left\{\left(s_{5.9565}\right),\left(s_{0}\right)\right\},\left\{\left(s_{5.9167}\right),\left(s_{0}\right)\right\},\left\{\left(s_{5.0769}\right),\left(s_{0.2542}\right)\right\},\left\{\left(s_{5.9802}\right),\left(s_{0}\right)\right\}]$$

$$H_{j^{-}}=[\left\{\left(s_{6}\right),\left(s_{0}\right)\right\},\left\{\left(s_{4.2857}\right),\left(s_{0.3333}\right)\right\},\left\{\left(s_{1.9459}\right),\left(s_{0.1791}\right)\right\},\left\{\left(s_{6}\right),\left(s_{0}\right)\right\},\left\{\left(s_{3.6923}\right),\left(s_{0.8824}\right)\right\}]$$

The distance between each alternative $A_{i}$ and $Hj^{+}$, and $Hj^{-}$ are calculated by using Equation (2.1). The $P_{i}^{+}$ and $P_{i}^{-}$, i=1,2,...,r are then computed as follows:

$$P_{i}^{+}=\overset{J}{\sum_{j=1}}w_{j}D\left(Hj^{+},H_{s_{ij}}\right),\;P_{i}^{-}=\overset{J}{\sum_{j=1}}w_{j}D\left(Hj^{-},H_{s_{ij}}\right)$$

The relative closeness (RC) coefficients for each alternative $A_{i}$ to the ideal solutions are calculated with the help of the following formula.

$$RC(A_{i})=\frac{P_{i}^{-}}{P_{i}^{+}+P_{i}^{-}}$$

The PIS, NIS and RC coefficients are calculated which can be seen in Table 8 along with the final ranking.

**Table 8:** PIS, NIS, RC coefficients and final ranking of alternatives using TOPSIS.

Alternatives	$P_{i}^{+}$	$P_{i}^{-}$	*RC*(*A*_*i*_)	Ranking
*A*_1_	0.0244	0.0229	0.4843	2
*A*_2_	0.0508	0.0284	0.3582	3
*A*_3_	0.0077	0.0447	0.8535	1
*A*_4_	0.0437	0.0242	0.3569	4

As shown in Table 7, the ranking results with respect to RC coefficients of all alternatives, i.e. $A_{3}≻A_{1}≻A_{2}≻A_{4}$ are matches with the EDAS method. This shows that our HIFLTS approach is also validated. DMs are given two types of linguistic articulations that are in support of and against alternatives such as HIFLTSs to perform distinct assessments under particular criteria. This can more accurately represent the fluffiness and vulnerability of experts. The proposed Einstein operational rules, as well as the HIFLEWA operator, are incredibly useful and viable for DMs when dealing with MCGDM situations that can demonstrate the predominance of the proposed strategy. The extension of the EDAS method with HIFLTSs can be useful for DMs in making decisions about the best option for a given set of criteria by using linguistic expressions correctly and effectively. As a result, the ranking results obtained using the EDAS technique are viable and reliable.

## 6 Conclusions

By employing the EDAS approach and proposing two equivalent transformation functions, a coordinated method for determining issues of a trading company in context of insurance policy in the best choice is proposed. The EDAS technique is a relatively new MCDM method with the most straightforward computation stages. As a result, it is more effectively applied to complicated challenges for the selection of best choices than any other MCDM technique. To overcome the DMs' hesitation, the EDAS approach was extended for HIFLTSs in this study. Many aggregation operators for HIFLTSs are proposed, and they are used to solve a real-world MCDM problem more effectively. Essential features of the hesitating intuitionistic fuzzy linguistic Einstein weighted averaging (HIFLEWA) operator are defined. Using the score function, we develop a hesitant intuitionistic fuzzy linguistic average matrix and average values for this matrix. Finally, a numerical example was presented to test the usefulness of EDAS method in ranked alternative. To understand how the new EDAS method presented itself, we conducted comparative analyses using the existing TOPSIS approach while evaluating the same numerical problem for alternative selection. The EDAS approach can be extended from specific angles in the future. (1) In the MCDM problem described above, the weights of the criteria are known. It is worthwhile to consider how to achieve the weight information of the criterion. (2) To demonstrate its appropriateness and proficiency, the EDAS technique will be applied to a growing number of real-life scenarios. (3) We will also look into the properties of the (HIFLEWA) operator and how they apply to different choice situations.

The main advantage of this study is the comprehensive platform and conversation it offers for comparison and innovation with related works and traditional methods. Numerous real-world scenarios offer potential applications for the proposed HIFLTS-based group decision-making method. For example, it could help with the development of systems that help decision-makers choose the best options. Similarly HIFLTS incorporates degrees of hesitation, it naturally offers a more expressive expression of doubt. This can be helpful in situations where decision-makers are reluctant to provide specific language information because they are uncertain as well. However, it is important to note that this paper has some drawbacks.

1. Based on the study analysis, it appears that the suggested model is more suited for challenges involving decision making of a small group of alternatives. When tackling complex issues involving GDM, it is crucial to look for ways to improve the effectiveness of the suggested models.
2. It is important to note that the mathematical description of HIFLTS appears to be more complex than other language models available in the literature. However, given the benefits of HIFLTS in helping DMs produce linguistic assessment values in an environment of uncertainty, HIFLTS deserves widespread recognition and additional study.
3. The proposed Einstein aggregation operators can consider the best aggregation, but there can be information loss throughout the aggregate process.
4. Unlike standard fuzzy sets, HIFLTS is a relatively new idea, and as such, there might not be many case studies or real-world applications showing its usefulness and applicability across a range of industries.

In the future, the proposed aggregation operations for HIFLTSs, which are compelling studies that deserve broader research, will allow us to further extend the ELECTRE and TODIM approaches to handle MCGDM problems. Furthermore, it is important to provide standardized procedures for operations (such as aggregation, defuzzification, and decision-making) within hesitant intuitionistic fuzzy sets. To ensure consistency and dependability in the usage of these approaches, benchmarks and comparative studies will be established and used to evaluate the performance of these methods in various applications.

## CRediT authorship contribution statement

**Shahzad Faizi:** Writing – review & editing, Writing – original draft, Visualization, Validation, Supervision, Software, Resources, Project administration, Methodology, Investigation, Funding acquisition, Formal analysis, Data curation, Conceptualization. **Wojciech Sałabun:** Writing – review & editing, Writing – original draft, Visualization, Validation, Supervision, Software, Resources, Project administration, Methodology, Investigation, Funding acquisition, Formal analysis, Data curation, Conceptualization. **Mubashar Shah:** Writing – review & editing, Writing – original draft, Visualization, Validation, Supervision, Software, Resources, Project administration, Methodology, Investigation, Funding acquisition, Formal analysis, Data curation, Conceptualization. **Tabasam Rashid:** Writing – review & editing, Writing – original draft, Visualization, Validation, Supervision, Software, Resources, Project administration, Methodology, Investigation, Funding acquisition, Formal analysis, Data curation, Conceptualization.

## Declaration of Competing Interest

The authors declare that they have no known competing financial interests or personal relationships that could have appeared to influence the work reported in this paper.
